# Associations of Medical Debt With Health Status, Premature Death, and Mortality in the US

**DOI:** 10.1001/jamanetworkopen.2023.54766

**Published:** 2024-03-04

**Authors:** Xuesong Han, Xin Hu, Zhiyuan Zheng, Kewei Sylvia Shi, K. Robin Yabroff

**Affiliations:** 1 Surveillance and Health Equity Science, American Cancer Society, Atlanta, Georgia; 2 Department of Public Health Sciences, University of Virginia School of Medicine, Charlottesville

## Abstract

**Importance:**

Medical debt is increasingly common in the US. Little is known regarding its association with population health.

**Objective:**

To examine the associations of medical debt with health status, premature death, and mortality at the county level in the US.

**Design, Setting, and Participants:**

This cross-sectional study was conducted at the US county level using 2018 medical debt data from the Urban Institute Debt in America project linked with 2018 data on self-reported health status and premature death from the County Health Rankings & Roadmaps and with 2015 to 2019 mortality data from the National Center for Health Statistics. Data analysis was performed from August 2022 to May 2023.

**Exposure:**

Share of population with any medical debt in collections and median amount of medical debt.

**Main Outcomes and Measures:**

Health status was measured as (1) the mean number of physically and mentally unhealthy days in the past 30 days per 1000 people, (2) the mean number of premature deaths measured as years of life lost before age 75 years per 1000 people, and (3) age-adjusted all-cause and 18 cause-specific mortality rates (eg, malignant cancers, heart disease, and suicide) per 100 000 person-years. Multivariable linear models were fitted to estimate the associations between medical debt and health outcomes.

**Results:**

A total of 2943 counties were included in this analysis. The median percentage of the county population aged 65 years or older was 18.3% (IQR, 15.8%-20.9%). Across counties, a median 3.0% (IQR, 1.2%-11.9%) of the population were Black residents, 4.3% (IQR, 2.3%-9.7%) were Hispanic residents, and 84.5% (IQR, 65.7%-93.3%) were White residents. On average, 19.8% (range, 0%-53.6%) of the population had medical debt. After adjusting for county-level sociodemographic characteristics, a 1–percentage point increase in the population with medical debt was associated with 18.3 (95% CI, 16.3-20.2) more physically unhealthy days and 17.9 (95% CI, 16.1-19.8) more mentally unhealthy days per 1000 people during the past month, 1.12 (95% CI, 1.03-1.21) years of life lost per 1000 people, and an increase of 7.51 (95% CI, 6.99-8.04) per 100 000 person-years in age-adjusted all-cause mortality rate. Associations of medical debt and elevated mortality rates were consistent for all leading causes of death, including cancer (1.12 [95% CI, 1.02-1.22]), heart disease (1.39 [95% CI, 1.21-1.57]), and suicide (0.09 [95% CI, 0.06-0.11]) per 100 000 person-years. Similar patterns were observed for associations between the median amount of medical debt and the aforementioned health outcomes.

**Conclusions and Relevance:**

These findings suggest that medical debt is associated with worse health status, more premature deaths, and higher mortality rates at the county level in the US. Therefore, policies increasing access to affordable health care, such as expanding health insurance coverage, may improve population health.

## Key Points

**Question:**

Is medical debt in collections associated with population health at the county level in the US?

**Findings:**

In this cross-sectional study of 2943 US counties, a higher share of the population with medical debt was associated with more days of poor physical and mental health, more years of life lost, and higher mortality rates for all-cause and leading causes of death following a dose-response association.

**Meaning:**

These findings suggest that medical debt is associated with worse population health and that policies increasing access to affordable health care, such as expanding health insurance coverage, may improve population health.

## Teaser

This cross-sectional study examines associations of medical debt with health status, premature death, and mortality among US counties.

## Introduction

Patients are increasingly burdened by high out-of-pocket costs for health care in the US,^1,2^ including problems paying medical bills and medical debt.^3,4,5,6,7,8^ A recent study of credit report data estimated that 17.8% of individuals had medical debt in collections in 2020.^9^

Certain populations are especially vulnerable to incurring medical debt. These populations include racial and ethnic minority individuals, female individuals, younger individuals, rural residents, those with multiple chronic diseases or serious psychological distress, uninsured individuals, those with high-deductible insurance plans, individuals with limited health insurance literacy, and individuals living in states that have not expanded Medicaid income eligibility under the Patient Protection and Affordable Care Act (ACA).^7,8,9,10,11,12,13,14,15,16,17,18,19,20^ Medical debt is adversely associated with multiple aspects of well-being, including delaying and forgoing recommended health care, prescription medication nonadherence,^21,22,23^ and food and housing insecurity.^4,24,25^ Through worsening of individual social risk factors and adversely affecting community social determinants of health, medical debt may exacerbate existing health disparities.^26^

Little is known about county-level associations of medical debt with population health, however. In this study, we linked the newly available medical debt information from the Urban Institute Debt in America project^27^ with health surveillance data to examine the associations of medical debt with health status, premature death, and mortality at the county level in the US.

## Methods

Because this cross-sectional study used publicly available and deidentified data, it was deemed exempt from institutional review board approval per the Common Rule (45 CFR §46) and informed consent requirements were waived. The study followed the Strengthening the Reporting of Observational Studies in Epidemiology (STROBE) reporting guideline.

### Data Source

Debt information was based on a 2% nationally representative panel of deidentified, consumer-level records from a major credit bureau in August 2018, collected through the Urban Institute Debt in America project.^28^ We used debt data aggregated at the county level. We extracted 2018 county-level statistics on self-reported health status and premature death from the County Health Rankings & Roadmaps compiled by the University of Wisconsin Population Health Institute from sources including the Behavioral Risk Factor Surveillance System and the National Center for Health Statistics.^29^ We obtained county-level, age-adjusted mortality rates from the National Center for Health Statistics for 2015 to 2019.^30^ County demographic and socioeconomic information for 2015 to 2019 was obtained from the US Census Bureau and the American Community Survey.^30^

### Measures

Medical debt in collections from the Debt in America project refers to medical debt sent to a third-party collector or assigned to a creditors’ internal collections department, in which the debt is normally 180 days or more past due and the creditor acted to collect the unpaid debt but was unsuccessful.^31^ Collections are defined as medical if the original creditor is identified as a medical practitioner or health care institution. Medical bills that are paid using a credit card or home refinance but ultimately go to collections would not appear as medical debt in collections.^31^ We examined 2 medical debt measures in analysis: the primary measure was the share (ie, percentage) of people with a credit bureau record who have any medical debt in collections; the secondary measure was the median amount of medical debt in 2018 US dollars among people with any medical debt in collections. These data are not reported when based on fewer than 50 people for a given county in the Debt in America project, so the secondary measure was only available for counties with 50 or more people in the sample with medical debt in collections. Any and median overall debt, which includes medical debt and other types of debt (eg, student loan debt in default and automobile or retail loan delinquency), were examined in supplementary analyses.

We examined 3 sets of health outcomes available in the aforementioned public data sources. These outcomes were as follows: (1) self-reported health status, including the age-adjusted mean numbers of physically unhealthy and mentally unhealthy days in the past 30 days per 1000 people in the county; (2) premature death, measured as age-adjusted years of potential life lost before age 75 years per 1000 people in the county, as used in the National Center for Health Statistics *Health, United States* reports^32^; and (3) age-adjusted all-cause mortality rate and cause-specific mortality rates for leading causes of death per 100 000 person-years in the county (eg, malignant cancers, heart disease, Alzheimer disease, diabetes, and suicide). For malignant cancers, we also examined the 5 most prevalent causes of cancer deaths separately (ie, lung and bronchus, colorectum, and pancreas among both sexes, breast cancer among women, and prostate cancer among men). Mortality data are based on the underlying cause of death and thus are mutually exclusive; the *International Classification of Diseases* codes for cause of death are available from the National Center for Health Statistics.^30^

We considered the following county-level sociodemographic characteristics as potential confounders for the associations between medical debt and health outcomes: percentages of the population who were non-Hispanic White (hereinafter, White) and had educational attainment of less than a high school diploma, uninsured, unemployed, and county’s metropolitan status. The percentage of the population with household income below the federal poverty line, which was highly correlated with the percentage of the population with education attainment of less than a high school diploma, was considered a potential mediator and thus was not controlled as a confounder. These characteristics were from US Census data based on self-reported information.^30^ Race and ethnicity was reported as American Indian or Alaska Native, Asian or Pacific Islander, Hispanic, non-Hispanic Black (hereinafter, Black), or White to describe the composition of the population. A full list of all study variables and corresponding data sources is provided in eTable 1 in Supplement 1.

### Statistical Analysis

We described county-level medical debt by county sociodemographic characteristics. Maps were created to show the geographic distribution of county-level medical debt in 2018. Scatterplots were generated to visualize the association between county-level medical debt and all-cause mortality. Bivariate and multivariable linear models were used to estimate the association between medical debt and health outcomes. All models included a random intercept for state to account for potential clustering effects at the state level and were weighted by county population size using the “weight” statement under the proc genmod procedure. Supplemental analyses were conducted by dividing debt variables into quartiles instead of a continuous scale in the models. Because mortality risk can vary by sex, we also examined the associations of medical debt and mortality rates in men and women separately. Similar supplemental analyses were conducted for overall debt. All statistical tests were 2 sided, with *P* < .05 considered statistically significant. Analysis was performed from August 2022 to May 2023 using SAS, version 9.4 (SAS Institute Inc).

## Results

A total of 2943 counties were included in this analysis (93% of all counties in the US). The median percentage of the county population aged 65 years or older was 18.3% (IQR, 15.8%-20.9%). Across counties, a median 0.4% (IQR, 0.3%-0.8%) of the population were American Indian or Alaska Native residents, 0.8% (IQR, 0.5%-1.6%) were Asian or Pacific Islander residents, 3.0% (IQR, 1.2%-11.9%) were Black residents, 4.3% (IQR, 2.3%-9.7%) were Hispanic residents, and 84.5% (IQR, 65.7%-93.3%) were White residents. A total of 1154 counties (39.2%) were in metropolitan areas. The average share of people with medical debt was 19.8%, ranging from 0% to 53.6% across counties. Counties with lower percentages of people who were White and higher percentages of people who were Black, with populations with low educational attainment, and with higher percentages of people with household income below the federal poverty level, uninsured, and unemployed tended to have a higher share of people with medical debt. Median medical debt had similar patterns (Table 1). The 994 counties excluded from the median medical debt analyses had a smaller population size, a smaller percentage of the population with any medical debt, and smaller percentages of racial and ethnic minority, unemployed, and uninsured individuals and were mostly nonmetropolitan counties (82.6%) compared with the 1934 included counties (eTable 2 in Supplement 1). Geographically, the counties with the highest medical debt burden were mostly in the South and Southwest areas, concentrated in the states of Texas, Louisiana, Georgia, Tennessee, South Carolina, North Carolina, and West Virginia (Figure).

**Table 1.** County-Level Medical Debt by Sociodemographic Characteristics in the US, 2018^a^

Characteristic^b^	Share with any medical debt, % (n = 2943)		Median medical debt, $ (n = 1949)	
	Mean (SD)	*P* value^c^	Mean (SD)	*P* value^c^
Overall	19.8 (9.4)	NA	751.5 (301.0)	NA
MSA status				
Metropolitan	19.0 (8.7)	<.001	715.2 (258.0)	<.001
Nonmetropolitan	20.3 (9.8)		788.4 (335.1)	
Race and ethnicity, quartile				
American Indian or Alaska Native				
1 (0.025-0.2579)	19.9 (8.7)	<.001	685.9 (283.9)	<.001
2 (0.258-0.40282)	21.1 (9.6)		745.3 (285.2)	
3 (0.40283-0.847)	20.6 (10.0)		782.2 (299.8)	
4 (0.848-84.855)	17.4 (9.0)		823.1 (329.9)	
Asian or Pacific Islander				
1 (0.067-0.4862)	21.4 (9.6)	<.001	741.2 (326.0)	<.001
2 (0.4863-0.757)	21.2 (9.8)		786.0 (311.6)	
3 (0.758-1.512)	20.3 (9.4)		783.4 (295.3)	
4 (1.513-69.786)	16.5 (8.0)		709.3 (281.0)	
Hispanic				
1 (0.577-2.308)	20.0 (9.1)	<.001	692.0 (318.3)	<.001
2 (2.309-4.286)	18.7 (9.3)		746.0 (297.1)	
3 (4.287-9.712)	19.2 (9.1)		760.6 (295.4)	
4 (9.713-96.262)	21.3 (10.0)		792.9 (288.9)	
Non-Hispanic Black				
1 (0.129-1.091)	15.6 (8.6)	<.001	782.4 (345.7)	.04
2 (1.092-2.709)	17.5 (8.7)		759.9 (331.8)	
3 (2.710-10.775)	20.7 (9.0)		725.1 (292.7)	
4 (10.776-85.962)	24.6 (8.8)		759.2 (263.1)	
Non-Hispanic White				
1 (2.822-65.609)	24.6 (9.9)	<.001	783.6 (275.7)	<.001
2 (65.610-84.892)	20.4 (8.9)		774.0 (296.5)	
3 (84.893-93.437)	16.4 (7.8)		729.3 (316.1)	
4 (93.438-98.390)	17.6 (8.8)		692.6 (316.3)	
Born outside the US, quartile				
1 (0.000-1.380)	20.5 (9.3)	.02	728.8 (334.1)	.01
2 (1.381-2.730)	19.7 (9.1)		745.1 (297.2)	
3 (2.731-5.670)	20.0 (9.4)		786.4 (295.3)	
4 (5.671-53.720)	19.0 (9.9)		737.5 (287.3)	
Age ≥65 y, quartile				
1 (3.200-15.800)	19.7 (9.6)	<.001	749.8 (272.1)	.15
2 (15.801-18.440)	21.3 (9.4)		735.7 (308.0)	
3 (18.441-21.220)	20.1 (9.3)		753.4 (321.7)	
4 (21.221-56.710)	17.8 (9.1)		785.9 (308.8)	
Educational attainment less than high school diploma, quartile				
1 (1.120-8.450)	12.1 (6.0)	<.001	701.5 (331.1)	<.001
2 (8.451-11.720)	17.1 (7.5)		705.1 (266.0)	
3 (11.721-16.650)	22.8 (7.9)		772.1 (288.6)	
4 (16.651-46.690)	26.7 (8.9)		813.8 (310.0)	
Household income below the federal poverty level, quartile				
1 (2.770-10.610)	13.2 (7.0)	<.001	674.5 (284.6)	<.001
2 (10.611-14.180)	17.3 (8.1)		753.0 (310.8)	
3 (14.181- 18.460)	22.2 (8.6)		773.5 (290.7)	
4 (18.461-55.450)	25.8 (8.7)		785.0 (305.2)	
Uninsured, quartile				
1 (2.105-7.311)	13.0 (7.4)	<.001	607.2 (283.5)	<.001
2 (7.312-10.315)	18.6 (8.0)		687.4 (257.7)	
3 (10.316-13.730)	22.0 (8.3)		794.5 (274.8)	
4 (13.731-43.798)	25.6 (9.1)		907.5 (309.5)	
Unemployed, quartile				
1 (0.000-3.620)	14.0 (8.7)	<.001	777.2 (332.3)	.001
2 (3.621-4.930)	18.5 (8.4)		718.5 (288.3)	
3 (4.931-6.480)	21.5 (8.4)		743.7 (289.9)	
4 (6.481-23.370)	24.4 (9.1)		785.2 (308.4)	

Abbreviations: MSA, metropolitan statistical area; NA, not applicable.

^a^ Data sources include Debt in America project of the Urban Institute and American Community Survey (eTable 1 in Supplement 1).

^b^ The ranges of percentages of population in each quarter of the counties are presented in the first column.

^c^ *P* values are from *t* tests.

**Figure.**
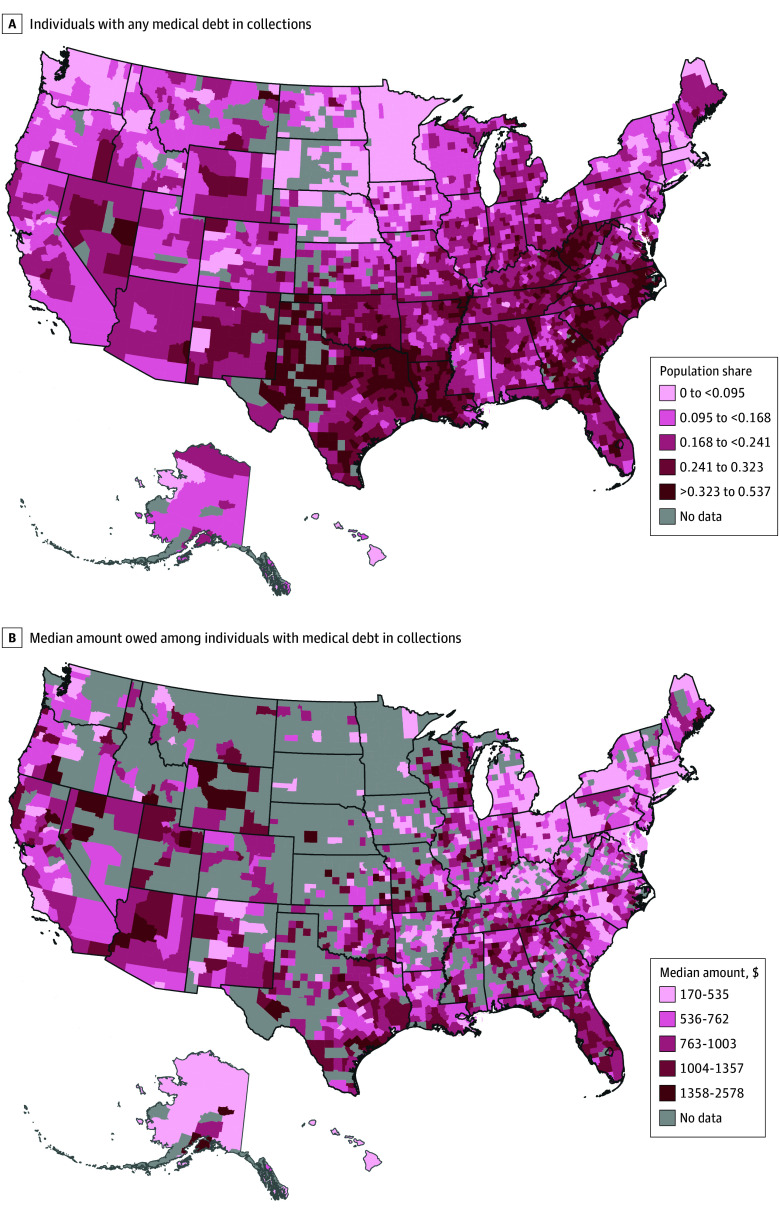
Medical Debt by US County, 2018 A, Share of population with any medical debt in collections. B, Median medical debt among people with any medical debt in collections. Data are from the Debt in America project of the Urban Institute.

On average, people in US counties experienced 4.4 physically unhealthy days and 4.7 mentally unhealthy days per person during the past 30 days and lost 85.2 years per 1000 people (or 31 days per person) due to premature death in 2018. After adjusting for county-level sociodemographic characteristics, a 1–percentage point increase in the population with medical debt was associated with 18.3 (95% CI, 16.3-20.2) more physically unhealthy days and 17.9 (95% CI, 16.1-19.8) more mentally unhealthy days in the past 30 days per 1000 people and with 1.12 (95% CI, 1.03-1.21) more years of life lost per 1000 people (Table 2).

**Table 2.** Association of County-Level Share of Population With Any Medical Debt With Health Status, Premature Death, and Age-Adjusted Mortality in the US^a^

Health outcome	No. of counties^b^	Mean (SD)	Crude model^c^		Adjusted model^d^	
			Coefficient	*P* value^e^	Coefficient	*P* value
Poor physical health during past 30 d, d/1000 people	2943	4413.2 (766.4)	51.5 (48.7-54.3)	<.001	18.3 (16.3-20.2)	<.001
Poor mental health during past 30 d, d/1000 people	2943	4702.2 (658.8)	41.1 (38.9-43.2)	<.001	17.9 (16.1-19.8)	<.001
Premature death, y lost/1000 people	2837	85.2 (25.3)	1.85 (1.76-1.95)	<.001	1.12 (1.03-1.21)	<.001
Age-adjusted mortality by cause of death, 100 000 person-years						
All cause	2943	824.6 (146.5)	11.57 (11.02-12.11)	<.001	7.51 (6.99-8.04)	<.001
Malignant cancers	2943	166.8 (27.4)	1.73 (1.63-1.83)	<.001	1.12 (1.02-1.22)	<.001
Lung and bronchus	2888	44.3 (12.9)	0.77 (0.72-0.81)	<.001	0.47 (0.43-0.52)	<.001
Prostate	2020	20.7 (6.5)	0.12 (0.09-0.15)	<.001	0.10 (0.07-0.13)	<.001
Female breast	2201	21.2 (5.3)	0.12 (0.10-0.14)	<.001	0.09 (0.07-0.11)	<.001
Colon and rectum	2538	15.8 (4.5)	0.17 (0.16-0.19)	<.001	0.09 (0.07-0.11)	<.001
Pancreas	2312	11.8 (2.8)	0.06 (0.05-0.07)	<.001	0.05 (0.04-0.06)	<.001
Heart disease	2943	187.1 (48.2)	2.72 (2.54-2.90)	<.001	1.39 (1.21-1.57)	<.001
Accidents and adverse effects	2917	58.4 (18.4)	0.96 (0.87-1.04)	<.001	0.48 (0.40-0.57)	<.001
Suicide and self-inflicted injury	2388	18.7 (7.1)	0.23 (0.20-0.26)	<.001	0.09 (0.06-0.11)	<.001
Chronic liver disease and cirrhosis	2227	13.6 (7.1)	0.29 (0.26-0.32)	<.001	0.13 (0.10-0.16)	<.001
Alzheimer disease	2741	34.1 (14.2)	0.29 (0.23-0.35)	<.001	0.26 (0.19-0.33)	<.001
Diabetes	2744	26.6 (12.1)	0.52 (0.47-0.56)	<.001	0.30 (0.25-0.35)	<.001
Chronic obstructive pulmonary disease and allied conditions	2921	52.1 (17.7)	1.20 (1.13-1.27)	<.001	0.71 (0.65-0.78)	<.001
Cerebrovascular diseases	2884	40.4 (10.6)	0.46 (0.42-0.50)	<.001	0.44 (0.39-0.49)	<.001
Homicide and legal intervention	1123	8.1 (6.6)	0.27 (0.23-0.32)	<.001	0.24 (0.20-0.28)	<.001
Septicemia	2244	13.5 (6.1)	0.24 (0.21-0.27)	<.001	0.14 (0.11-0.17)	<.001
Nephritis, nephrotic syndrome, and nephrosis	2436	16.6 (7.2)	0.29 (0.26-0.32)	<.001	0.19 (0.16-0.22)	<.001
Hypertension without heart disease	1973	10.6 (6.8)	0.15 (0.12-0.17)	<.001	0.09 (0.06-0.12)	<.001

^a^ Data sources include the Urban Institute Debt in America project, the American Community Survey, the Behavioral Risk Factor Surveillance System, and the National Center for Health Statistics (eTable 1 in Supplement 1).

^b^ Varies by health outcomes because county-level health statistics were not provided if there were fewer than 10 cases.

^c^ Crude models were weighted by population size, with SEs clustered at the state level.

^d^ Controlled for county-level percentages of the population who were non-Hispanic White, had educational attainment less than a high school diploma, were uninsured, or were unemployed and for metropolitan statistical area status and were weighted by population size, with SEs clustered at the state level.

^e^ *P* values are from *t* tests.

The mean (SD) county-level, age-adjusted all-cause mortality rate was 824.6 (146.5) per 100 000 person-years in 2015 to 2019. The scatterplot showed a clear dose-response association between medical debt and mortality rate (eFigure 2A in Supplement 1). After adjusting for county-level sociodemographic characteristics, a 1–percentage point increase in the population with medical debt was associated with an increase in the all-cause mortality rate of 7.51 (95% CI, 6.99-8.04) per 100 000 person-years (Table 2). In terms of cause-specific mortality rates, a higher share of the population with medical debt was associated with higher mortality rates for all causes examined. For example, a 1–percentage point increase in the population with medical debt was associated with increases of 1.39 (95% CI, 1.21-1.57), 1.12 (95% CI, 1.02-1.22), 0.71 (95% CI, 0.65-0.78), 0.30 (95% CI, 0.25-0.35), and 0.09 (95% CI, 0.06-0.11) per 100 000 person-years in mortality rates of heart disease, malignant cancers, chronic obstructive pulmonary disease, diabetes, and suicide, respectively (Table 2).

Among the 1949 counties with median medical debt information available (ie, ≥50 people with any medical debt in collections in the sample), every $100 increase in median medical debt was associated with 8.0 (95% CI, 1.2-14.9) more physically unhealthy days and 6.8 (95% CI, 0.2-13.3) more mentally unhealthy days in the past 30 days per 1000 people, with 0.64 (95% CI, 0.33-0.96) more years of life lost per 1000 people, and with an increase of 4.81 (95% CI, 2.87-6.75) per 100 000 person-years in all-cause mortality rate, with a dose-response association (Table 3 and eFigure 2B in Supplement 1). Median medical debt was also positively associated with cause-specific mortality rates, with associations observed for 12 of 18 causes of death examined (Table 3). When stratified by sex, the positive associations between medical debt and mortality rates were consistently seen in both sexes, and the magnitude appeared to be generally larger in men than in women (eTable 3 in Supplement 1).

**Table 3.** Association of County-Level Median Medical Debt With Health Status, Premature Death, and Age-Adjusted Mortality in the US^a^

Health outcome	No. of counties^b^	Mean (SD)	Crude model^c^		Adjusted model^d^	
			Coefficient	*P* value^e^	Coefficient	*P* value
Poor physical health during past 30 d, d/1000 people	1949	4479.3 (703.8)	52.3 (40.0 to 64.7)	<.001	8.0 (1.2 to 14.9)	.02
Poor mental health during past 30 d, d/1000 people	1949	4803.6 (592.8)	42.8 (33.2 to 52.5)	<.001	6.8 (0.2 to 13.3)	.04
Premature death, y lost/1000 people	1949	85.4 (23.4)	1.70 (1.29 to 2.12)	<.001	0.64 (0.33 to 0.96)	<.001
Age-adjusted mortality by cause of death, 100 000 person-years						
All cause	1949	835.5 (139.5)	11.70 (9.23 to 14.18)	<.001	4.81 (2.87 to 6.75)	<.001
Malignant cancers	1949	168.2 (24.4)	1.96 (1.55 to 2.38)	<.001	0.86 (0.52 to 1.21)	<.001
Lung and bronchus	1947	44.8 (12.3)	0.93 (0.74 to 1.12)	<.001	0.33 (0.18 to 0.47)	<.001
Prostate	1730	20.1 (5.7)	0.07 (−0.02 to 0.17)	.13	0.10 (0.01 to 0.19)	.03
Female breast	1847	20.9 (4.7)	0.10 (0.03 to 0.17)	.006	0.08 (0.01 to 0.15)	.02
Colon and rectum	1933	15.3 (3.8)	0.17 (0.11 to 0.22)	<.001	0.06 (0.01 to 0.11)	.02
Pancreas	1869	11.5 (2.4)	0.03 (−0.002 to 0.07)	.06	0.03 (−0.01 to 0.06)	.15
Heart disease	1949	190.4 (46.2)	2.52 (1.78 to 3.26)	<.001	0.63 (0.01 to 1.25)	.045
Accidents and adverse effects	1949	57.7 (17.3)	0.89 (0.58 to 1.21)	<.001	0.08 (−0.19 to 0.36)	.54
Suicide and self-inflicted injury	1881	17.5 (5.5)	0.47 (0.38 to 0.57)	<.001	0.13 (0.06 to 0.20)	<.001
Chronic liver disease and cirrhosis	1850	13.1 (6.1)	0.30 (0.20 to 0.39)	<.001	0.02 (−0.06 to 0.11)	.56
Alzheimer disease	1938	34.5 (13.7)	0.42 (0.21 to 0.64)	<.001	0.21 (−0.01 to 0.42)	.06
Diabetes	1944	25.7 (11.5)	0.50 (0.32 to 0.68)	<.001	0.29 (0.12 to 0.45)	<.001
Chronic obstructive pulmonary disease and allied conditions	1948	52.5 (17.5)	1.51 (1.22 to 1.80)	<.001	0.43 (0.21 to 0.65)	<.001
Cerebrovascular diseases	1949	40.9 (9.9)	0.38 (0.21 to 0.55)	<.001	0.28 (0.11 to 0.45)	.001
Homicide and legal intervention	1084	7.7 (6.1)	0.06 (−0.11 to 0.23)	.51	0.12 (−0.02 to 0.25)	.08
Septicemia	1868	13.3 (5.8)	0.24 (0.14 to 0.33)	<.001	0.15 (0.06 to 0.24)	.001
Nephritis, nephrotic syndrome, and nephrosis	1916	16.3 (6.5)	0.30 (0.20 to 0.40)	<.001	0.18 (0.08 to 0.27)	<.001
Hypertension without heart disease	1685	9.8 (5.7)	0.04 (−0.05 to 0.14)	.36	0.05 (−0.04 to 0.14)	.28

^a^ Data are presented as 2018 hundred US dollars. Sources include the Urban Institute Debt in America project, the American Community Survey, the Behavioral Risk Factor Surveillance System, and the National Center for Health Statistics (eTable 1 in Supplement 1).

^b^ Varies by health outcomes because county-level health statistics were not provided if there were fewer than 10 cases.

^c^ Weighted by population size, with SEs clustered at the state level.

^d^ Controlled for county-level percentages of the population who were non-Hispanic White, had educational attainment less than a high school diploma, were uninsured, or were unemployed and for metropolitan statistical area status and were weighted by population size, with SEs clustered at the state level.

^e^ *P* values are from *t* tests.

Supplemental analyses with quartile medical debt variables showed a similar dose-response association between medical debt and health outcomes (eTables 4 and 5 in Supplement 1). Supplemental analyses for share of overall debt and median overall debt had the same patterns, with larger associations (eTables 6-11 and eFigures 1, 2C, and 2D in Supplement 1).

## Discussion

Using nationwide county-level medical debt data linked with health surveillance data, we observed consistent associations between medical debt and worse population health across multiple measures, including self-reported unhealthy days, years of life lost due to premature death, and all-cause and cause-specific mortality rates. Along with accumulating individual-level evidence that medical financial hardship is associated with delayed health care and worse health outcomes,^6,33,34,35^ our findings reinforce medical debt as an important social determinant of health, which may threaten public health in the US.

Policies expanding insurance coverage have been shown to be effective in mitigating medical debt. As reported by Kluender et al,^9^ states that expanded Medicaid income eligibility as part of the ACA experienced a 34.0–percentage point greater decline on average in medical debt incurred during the last year (from $330 to $175) than the states that did not expand Medicaid (from $613 to $550). Kluender et al^9^ also reported a decrease in disparities in medical debt by area-level income in Medicaid expansion states but increased disparities in nonexpansion states. In a single-state study conducted among individuals gaining Medicaid coverage through Louisiana Medicaid expansion in 2016, medical debt in collections declined by 13.5% by 2019.^20^ Of note, in our study, the states with the highest share of medical debt are also the states that did not expand Medicaid income eligibility. Although the ACA led to historical progress in reducing uninsurance and improving care access, recent challenges may reverse these gains. For example, adding work requirements for Medicaid^36^ coverage may increase the risk of losing insurance and accumulating medical debt. Actions to undermine provisions of the ACA that improve coverage and access would likely widen the geographic disparities in medical debt as we observed and would jeopardize population health in the Southern states the most, which already bear the highest medical debt and mortality rates. Moreover, despite recent evidence that Medicaid expansion was associated with reduced mortality rates,^37^ the contribution of mitigated medical debt is unknown and warrants future investigation.

Several recent federal policies may help alleviate medical debt in the coming years. The Consumer Financial Protection Bureau debt collection final rule, implemented in November 2021, prohibits debt collectors from furnishing collection information to a consumer reporting company before communicating with the debtor, a practice previously used by some medical debt collectors.^23^ The rule also requires debt collectors to provide an itemization of the debt, which may help debtors recognize and understand medical debts in collection.^23^ The No Surprises Act, designed to protect consumers from unexpected medical bills, took effect in January 2022.^23^ The Inflation Reduction Act, signed into law in August 2022, includes provisions intended to lower out-of-pocket costs for prescription drugs and extend ACA subsidies. In April 2023, the 3 nationwide consumer reporting companies (Equifax, Experian, and TransUnion) announced that they removed unpaid medical collections under $500 from consumer credit reports.^38^ How and to what extent these policies and changes in consumer credit reports will affect the growing medical debt burden warrants future investigation.

For working-age Americans, employer-sponsored insurance is the primary source of health insurance coverage.^39^ In addition to offering health insurance, employers can also reduce the burden of medical debt for workers by providing paid sick leave and health savings account benefits when offering high-deductible health plans. Previous research shows that employees without these benefits were more likely to forgo or delay care and skip preventive services,^40,41^ which could lead to worse disease outcomes and higher medical costs. Moreover, without paid sick leave, some individuals may delay or forgo health care when a life-threatening disease is diagnosed if they cannot afford lost wages. Extended unpaid absences from work may result in job loss and loss of employer-sponsored health insurance coverage.

Hospitals can play a key role in the incurrence and collection of medical debt. A recent study from the Urban Institute showed that nearly three-quarters of adults with past-due medical debt reported owing some of that debt to hospitals.^42^ Although the ACA requires nonprofit hospitals to have written financial assistance policies and limit charges and collections against patients screened as eligible for financial assistance, the rules are underenforced and do not apply to for-profit or government-run hospitals.^43^ Some states, such as California, Maryland, New Mexico, and Washington, have enacted new legislation to extend the federal protection for those with medical debt by expanding the financial assistance policies to all types of hospitals, establishing clear eligibility criteria for charity care, and imposing stricter regulation to hospitals on medical debt collection.^23,42,43^ In addition to financial assistance programs, hospitals and health care systems can also help alleviate medical debt by strengthening cost-related communication with patients for informed decision-making on care and payment planning and by developing or adopting screening tools to proactively identify and support patients with multiple health-related social needs (eg, food and housing insecurity, transportation barriers to care) who may have a higher risk of medical debt. Increasing attention to identifying patients with health-related social needs in clinical settings and connecting them with services, such as through the Centers for Medicare & Medicaid Services Enhancing Oncology Model,^44^ may also reduce the risk of medical debt.

### Limitations

Our study has some limitations. First, with a 2% nationally representative sample of consumers from credit reports, medical debt estimates (especially the median medical debt measure) were missing for some less populous counties. Moreover, the debt information may not be representative of less populous counties. Therefore, we weighted our analyses by county population to minimize the potential bias from the smaller counties. Second, we were not able to examine the source of medical debt (eg, hospitalization, prescription medication) due to lack of information. Moreover, the calculation of the medical debt measures could not account for those individuals not in the credit system, such as immigrants who are undocumented and some people with an incarceration history, who may be financially disadvantaged and face more health challenges.^45^ Therefore, our estimates of the association between county-level medical debt and health outcomes are likely understated. As with any observational study, we measured associations that cannot be interpreted causally. Nevertheless, our findings of the associations between medical debt and public health surveillance measures fill a critical gap in the literature, and they can inform policy makers and public health advocates.

We used pre–COVID-19 pandemic debt and health data to avoid confounding from the pandemic, which exacerbated medical debt and deterioration of population health. As the COVID-19 public health emergency ends and associated financial assistance and insurance enrollment policies expire,^46^ future research is warranted to examine the effects of the pandemic and associated policies on medical debt and population health. (Such policies include Medicaid unwinding, as the provision of continuous enrollment in Medicaid under the Families First Coronavirus Response Act expired on March 31, 2023, leading to millions of people losing Medicaid coverage as states redetermined eligibility for all Medicaid enrollees and disenrolled those no longer eligible or whose eligibility could not be determined.) Finally, we observed a similar, even larger, association between overall debt and health outcomes at the county level, although this was not a focus of our study. This finding suggests that policies and programs addressing the burden from other debts such as student loans may help improve population health as well.

## Conclusions

By linking recent nationwide county-level medical debt data with health surveillance data in this cross-sectional study, we observed associations between medical debt and worse health outcomes, including more unhealthy days, more premature deaths, and higher mortality rates. These findings suggest that efforts from multiple stakeholders, including federal, state, hospital and health care systems, and employers, to address medical debt may also improve population health in the US.

## Notes

Supplement 1. **eTable 1.** Study Variables and Data Sources **eTable 2.** Sample Characteristics Comparison Between Counties With Nonmissing Median Medical Debt and With Missing Median Medical Debt **eTable 3.** Association of County-Level Medical Debt and Age-Adjusted Mortality Rate (per 100 000 Person-Years) by Sex in the US **eTable 4.** Association of Quartiles of County-Level Share of Population With Any Medical Debt With Health Status, Premature Death, and Age-Adjusted Mortality in the US **eTable 5.** Association of Quartiles of County-Level Median Medical Debt With Health Status, Premature Death, and Age-Adjusted Mortality in the US **eTable 6.** County-Level Debt by Sociodemographic Characteristics in the US, 2018 **eTable 7.** Association of County-Level Share of Population With Any Debt With Health Status, Premature Death, and Age-Adjusted Mortality in the US **eTable 8.** Association of County-Level Median Debt (in 2018 Hundred Dollars) With Health Status, Premature Death, and Age-Adjusted Mortality in the US **eTable 9.** Association of County-Level Debt and Age-Adjusted Mortality Rate (per 100 000 Person-Years) by Sex in the US **eTable 10.** Association of Quartiles of County-Level Share of Population With Any Debt With Health Status, Premature Death, and Age-Adjusted Mortality in the US **eTable 11.** Association of Quartiles of County-Level Median Debt With Health Status, Premature Death, and Age-Adjusted Mortality in the US **eFigure 1.** Medical Debt by County in the US, 2018 **eFigure 2.** County-Level Debt and Age-Adjusted All-Cause Mortality in the US

Supplement 2.
Data Sharing Statement
