# Supplementary material for: Associations of Medical Debt With Health Status, Premature Death, and Mortality in the US
Source: JAMA Netw Open. 2024 Mar 4;7(3):e2354766. doi: 10.1001/jamanetworkopen.2023.54766 (PMC10912961; doi:10.1001/jamanetworkopen.2023.54766)
Supplement: Supplement 1. — eTable 1. Study Variables and Data Sources eTable 2. Sample Characteristics Comparison Between Counties With Nonmissing Median Medical Debt and With Missing Median Medical Debt eTable 3. Association of County-Level Medical Debt and Age-Adjusted Mortality Rate (per 100 000 Person-Years) by Sex in the US eTable 4. Association of Quartiles of County-Level Share of Population With Any Medical Debt With Health Status, Premature Death, and Age-Adjusted Mortality in the US eTable 5. Association of Quartiles of County-Level Median Medical Debt With Health Status, Premature Death, and Age-Adjusted Mortality in the US eTable 6. County-Level Debt by Sociodemographic Characteristics in the US, 2018 eTable 7. Association of County-Level Share of Population With Any Debt With Health Status, Premature Death, and Age-Adjusted Mortality in the US eTable 8. Association of County-Level Median Debt (in 2018 Hundred Dollars) With Health Status, Premature Death, and Age-Adjusted Mortality in the US eTable 9. Association of County-Level Debt and Age-Adjusted Mortality Rate (per 100 000 Person-Years) by Sex in the US eTable 10. Association of Quartiles of County-Level Share of Population With Any Debt With Health Status, Premature Death, and Age-Adjusted Mortality in the US eTable 11. Association of Quartiles of County-Level Median Debt With Health Status, Premature Death, and Age-Adjusted Mortality in the US eFigure 1. Medical Debt by County in the US, 2018 eFigure 2. County-Level Debt and Age-Adjusted All-Cause Mortality in the US [file jamanetwopen-e2354766-s001.pdf]

Supplementary Online Content

Han X, Hu X, Zheng Z, Shi KS, Yabroff KR. Associations of medical debt with health status, premature death, and mortality in the US. *JAMA Netw Open*. 2024;7(3):e2354766. doi:10.1001/jamanetworkopen.2023.54766

eTable 1. Study Variables and Data Sources

eTable 2. Sample Characteristics Comparison Between Counties With Nonmissing Median Medical Debt and With Missing Median Medical Debt

eTable 3. Association of County-Level Medical Debt and Age-Adjusted Mortality Rate (per 100 000 Person-Years) by Sex in the US

eTable 4. Association of Quartiles of County-Level Share of Population With Any Medical Debt With Health Status, Premature Death, and Age-Adjusted Mortality in the US

eTable 5. Association of Quartiles of County-Level Median Medical Debt With Health Status, Premature Death, and Age-Adjusted Mortality in the US

eTable 6. County-Level Debt by Sociodemographic Characteristics in the US, 2018

eTable 7. Association of County-Level Share of Population With Any Debt With Health Status, Premature Death, and Age-Adjusted Mortality in the US

eTable 8. Association of County-Level Median Debt (in 2018 Hundred Dollars) With Health Status, Premature Death, and Age-Adjusted Mortality in the US

eTable 9. Association of County-Level Debt and Age-Adjusted Mortality Rate (per 100 000 Person-Years) by Sex in the US

eTable 10. Association of Quartiles of County-Level Share of Population With Any Debt With Health Status, Premature Death, and Age-Adjusted Mortality in the US

eTable 11. Association of Quartiles of County-Level Median Debt With Health Status, Premature Death, and Age-Adjusted Mortality in the US

eFigure 1. Medical Debt by County in the US, 2018

eFigure 2. County-Level Debt and Age-Adjusted All-Cause Mortality in the US

This supplementary material has been provided by the authors to give readers additional information about their work.

© 2024 Han X et al. *JAMA Network Open.*

eTable 1. Study Variables and Data Sources

Variable	Description	Data source	Year of data	Access
Share of population with any medical debt in collection		Debt in American Project, Urban Institute	2018	Email request to externalaffairs@urban.org
Median medical debt		Debt in American Project, Urban Institute	2018	Email request to externalaffairs@urban.org
Percent of Non-Hispanic White		U.S. Census Bureau	2015-2019	SEER*Stat
Percent of Non-Hispanic Black		U.S. Census Bureau	2015-2019	SEER*Stat
Percent of Hispanic		U.S. Census Bureau	2015-2019	SEER*Stat
Percent of Non-Hispanic Asian Pacific Islander		U.S. Census Bureau	2015-2019	SEER*Stat
Percent of Non-Hispanic American Indian and Alaska Native		U.S. Census Bureau	2015-2019	SEER*Stat
Percent of Foreign Born		American Community Survey	2015-2019	SEER*Stat
Percent of 65 Years or Older		American Community Survey	2015-2019	SEER*Stat
Percent of Below Poverty Level		American Community Survey	2015-2019	SEER*Stat
Percent of population with less than high school education		American Community Survey	2015-2019	SEER*Stat
Metropolitan status		Rural-Urban Continuum Codes developed by the United States Department of Agriculture (USDA)	2013	SEER*Stat
Uninsured rate		Debt in American Project, Urban Institute: American Community Survey	2017	Email request to externalaffairs@urban.org
Unemployment rate		American Community Survey	2015-2019	SEER*Stat
Poor physical health days	Average number of physically unhealthy days reported in past 30 days (age-adjusted).	County Health Ranking: Behavioral Risk Factor Surveillance System	2018	https://www.countyhealthrankings.org/
Poor mental health days	Average number of mentally unhealthy days reported in past 30 days (age-adjusted).	County Health Ranking: Behavioral Risk Factor Surveillance System	2018	https://www.countyhealthrankings.org/
Premature death	Years of potential life lost before age 75 per 100,000 population (age-adjusted).	County Health Ranking: National Center for Health Statistics - Mortality Files	2017-2019	https://www.countyhealthrankings.org/
Age-adjusted mortality rate	Number of deaths per 100,000 person-years	National Center for Health Statistics	2015-2019	SEER*Stat

eTable 2. Sample Characteristics Comparison Between Counties With Nonmissing Median Medical Debt and With Missing Median Medical Debt

	Non-Missing		Missing		P-value[1]
	N	%	N	%	
**Total N**	**1949**		**994**		
**Population, Mean (STD)**	797454	(SD=2051983)	67335	(SD=68686)	<.001
**Percent of population with any medical debt, Mean (STD)**	22.1%	(SD=8.7%)	15.3%	(SD=9.1%)	<.001
**MSA**					<.001
**Metropolitan**	981	50.3%	173	17.4%	
**Non-metropolitan**	968	49.7%	821	82.6%	
**% AIAN**					<.001
**Q1: 0.025-0.2579**	562	28.8%	198	19.9%	
**Q2: 0.2580-0.40282**	551	28.3%	215	21.6%	
**Q3: 0.40283-0.847**	476	24.4%	247	24.8%	
**Q4: 0.848-84.855**	360	18.5%	334	33.6%	
**% API**					<.001
**Q1: 0.067-0.4862**	310	15.9%	366	36.8%	
**Q2: 0.4863-0.757**	426	21.9%	318	32.0%	
**Q3: 0.758-1.512**	537	27.6%	220	22.1%	
**Q4: 1.513-69.786**	676	34.7%	90	9.1%	
**% Hispanic**					<.001
**Q1: 0.577-2.308**	419	21.5%	313	31.5%	
**Q2: 2.309-4.286**	447	22.9%	298	30.0%	
**Q3: 4.287-9.712**	537	27.6%	201	20.2%	
**Q4: 9.713-96.262**	546	28.0%	182	18.3%	
**% NH Black**					<.001
**Q1: 0.129-1.091**	263	13.5%	418	42.1%	
**Q2: 1.092-2.709**	431	22.1%	291	29.3%	
**Q3: 2.710-10.775**	626	32.1%	140	14.1%	
**Q4: 10.776-85.962**	629	32.3%	145	14.6%	
**% NH White**					<.001
**Q1: 2.822-65.609**	554	28.4%	180	18.1%	
**Q2: 65.610-84.892**	586	30.1%	172	17.3%	
**Q3: 84.893-93.437**	456	23.4%	279	28.1%	
**Q4: 93.438-98.390**	353	18.1%	363	36.5%	
**% Foreign born**					<.001
**Q1: 0.000-1.380**	335	17.2%	375	37.7%	
**Q2: 1.381-2.730**	466	23.9%	275	27.7%	
**Q3: 2.731-5.670**	547	28.1%	200	20.1%	
**Q4: 5.671-53.720**	601	30.8%	144	14.5%	
**% 65 years or older**					<.001
**Q1: 3.200-15.800**	611	31.3%	142	14.3%	
**Q2: 15.801-18.440**	594	30.5%	175	17.6%	

© 2024 Han X et al. *JAMA Network Open.*

**Q3: 18.441-21.220**	466	23.9%	292	29.4%	
**Q4: 21.221-56.710**	278	14.3%	385	38.7%	
**% Less than high school graduate**					<.001
**Q1: 1.120-8.450**	387	19.9%	316	31.8%	
**Q2: 8.451-11.720**	498	25.6%	252	25.4%	
**Q3: 11.721-16.650**	571	29.3%	180	18.1%	
**Q4: 16.651-46.690**	493	25.3%	246	24.7%	
**% Below poverty**					<.001
**Q1: 2.770-10.610**	396	20.3%	296	29.8%	
**Q2: 10.611-14.180**	471	24.2%	269	27.1%	
**Q3: 14.181-18.460**	554	28.4%	204	20.5%	
**Q4: 18.461-55.450**	528	27.1%	225	22.6%	
**% Uninsured**					<.001
**Q1: 2.105-7.311**	421	21.6%	320	32.2%	
**Q2: 7.312-10.315**	528	27.1%	214	21.5%	
**Q3: 10.316-13.730**	543	27.9%	205	20.6%	
**Q4: 13.731-43.798**	457	23.4%	255	25.7%	
**% Unemployed**					<.001
**Q1: 0.000-3.620**	242	12.4%	424	42.7%	
**Q2: 3.621-4.930**	569	29.2%	190	19.1%	
**Q3: 4.931-6.480**	619	31.8%	156	15.7%	
**Q4: 6.481-23.370**	519	26.6%	224	22.5%	

Data sources include Debt in America Project of the Urban Institute and American Community Survey (Appendix Table 1).

Abbreviations: MSA: metropolitan statistical area; NH: non-Hispanic; API= Asian pacific islander; AIAN=American Indian and Alaska Native; Q: quartile; SD: standard deviation.

The range of percentages in each quarter of the counties are presented in 1st column.

1 P-values are from T-tests for debt variables and Chi-square tests for other categorical variables.

© 2024 Han X et al. *JAMA Network Open.*

eTable 3. Association of County-Level Medical Debt and Age-Adjusted Mortality Rate (per 100 000 Person-Years) by Sex in the US

	Any medical debt				Median medical debt (2018 USD $100)			
	Men		Women		Men		Women	
Cause of mortality	Coefficient	P-value[1]	Coefficient	P-value[1]	Coefficient	P-value[1]	Coefficient	P-value[1]
**All cause**	**9.20 (8.52, 9.88)**	**<.001**	**6.36 (5.91, 6.81)**	**<.001**	**5.55 (3.06, 8.04)**	**<.001**	**4.39 (2.75, 6.04)**	**<.001**
**Malignant cancers**	**1.54 (1.40, 1.68)**	**<.001**	**0.84 (0.75, 0.94)**	**<.001**	**1.16 (0.68, 1.64)**	**<.001**	**0.65 (0.35, 0.96)**	**<.001**
**Lung and bronchus**	**0.63 (0.57, 0.70)**	**<.001**	**0.36 (0.32, 0.41)**	**<.001**	**0.38 (0.18, 0.59)**	**<0.001**	**0.28 (0.14, 0.41)**	**<.001**
**Prostate**	**0.11 (0.08, 0.14)**	**<.001**	-	**<.001**	**0.12 (0.03, 0.21)**	**0.011**	-	-
**Female breast**	-	**<.001**	**0.09 (0.06, 0.11)**	**<.001**	-	-	**0.07 (0.002, 0.14)**	**0.043**
**Colon and rectum**	**0.12 (0.09, 0.14)**	**<.001**	**0.07 (0.05, 0.09)**	**<.001**	0.06 (-0.01, 0.13)	0.12	**0.06 (0.003, 0.11)**	**0.039**
**Pancreas**	**9.20 (8.52, 9.88)**	**<.001**	**0.04 (0.03, 0.06)**	**<.001**	0.05 (-0.01, 0.10)	0.08	0.01 (-0.04, 0.05)	0.73
**Heart disease**	**1.84 (1.61, 2.07)**	**<.001**	**1.11 (0.96, 1.27)**	**<.001**	0.73 (-0.06, 1.52)	0.07	**0.56 (0.02, 1.10)**	**0.042**
**Accidents and adverse effects**	**0.59 (0.47, 0.71)**	**<.001**	**0.35 (0.28, 0.41)**	**<.001**	-0.01 (-0.40, 0.37)	0.95	**0.20 (0.01, 0.40)**	**0.043**
**Suicide and self-inflicted injury**	**0.16 (0.12, 0.20)**	**<.001**	**0.03 (0.01, 0.05)**	**0.014**	**0.18 (0.06, 0.29)**	**0.002**	**0.08 (0.02, 0.15)**	**0.011**
**Chronic liver disease and cirrhosis**	**0.16 (0.12, 0.21)**	**<.001**	**0.08 (0.05, 0.12)**	**<.001**	0.02 (-0.10, 0.14)	0.73	0.03 (-0.06, 0.11)	0.57
**Alzheimer disease**	**0.23 (0.17, 0.30)**	**<.001**	**0.27 (0.20, 0.35)**	**<.001**	0.17 (-0.03, 0.37)	0.09	0.24 (-0.01, 0.49)	0.06
**Diabetes**	**0.35 (0.28, 0.41)**	**<.001**	**0.27 (0.22, 0.32)**	**<.001**	**0.37 (0.17, 0.58)**	**<0.001**	**0.23 (0.09, 0.38)**	**0.002**
**Chronic obstructive pulmonary disease and allied conditions**	**0.82 (0.74, 0.90)**	**<.001**	**0.68 (0.62, 0.75)**	**<.001**	**0.48 (0.21, 0.74)**	**<0.001**	**0.39 (0.17, 0.61)**	**<0.001**
**Cerebrovascular diseases**	**0.49 (0.43, 0.55)**	**<.001**	**0.42 (0.37, 0.48)**	**<.001**	**0.37 (0.18, 0.56)**	**<0.001**	**0.24 (0.06, 0.42)**	**0.009**
**Homicide and legal intervention**	**0.41 (0.32, 0.49)**	**<.001**	**0.09 (0.07, 0.11)**	**<.001**	0.22 (-0.06, 0.50)	0.12	**0.08 (0.01, 0.15)**	**0.023**
**Septicemia**	**0.15 (0.11, 0.20)**	**<.001**	**0.12 (0.09, 0.15)**	**<.001**	**0.15 (0.03, 0.27)**	**0.015**	**0.15 (0.05, 0.24)**	**0.003**
**Nephritis, nephrotic syndrome, and nephrosis**	**0.21 (0.16, 0.25)**	**<.00**	**0.18 (0.15, 0.21)**	**<.001**	**0.19 (0.06, 0.32)**	**0.004**	**0.16 (0.07, 0.26)**	**0.001**
**Hypertension without heart disease**	**0.10 (0.05, 0.14)**	**<.0001**	**0.08 (0.05, 0.12)**	**<.001**	0.11 (-0.02, 0.24)	0.10	0.03 (-0.07, 0.13)	0.53

Data sources include Debt in America Project of the Urban Institute, American Community Survey, Behavioral Risk Factor Surveillance System, and National Center for Health Statistics (Appendix Table 1).

Models controlled for county-level % of non-Hispanic White, % less than high school graduate, % uninsured, % unemployed, and metropolitan statistical area status. All models were weighted by population size, with standard errors clustered at the state level.

[1] P-values are from T-tests.

© 2024 Han X et al. *JAMA Network Open.*

eTable 4. Association of Quartiles of County-Level Share of Population With Any Medical Debt With Health Status, Premature Death, and Age-Adjusted Mortality in the US

Health outcome	Q2 (vs. Q1)		Q3 (vs. Q1)		Q4 (vs. Q1)	
	Coefficient	P-value[1]	Coefficient	P-value[1]	Coefficient	P-value[1]
**Poor physical health during past 30 d, d/1000 people**	**148.95 (116.27, 181.62)**	**<.001**	**311.68 (270.49, 352.87)**	**<.001**	**430.73 (373.93, 487.53)**	**<.001**
**Poor mental health during past 30 d, d/1000 people**	**195.02 (164.73, 225.30)**	**<.001**	**384.48 (346.29, 422.68)**	**<.001**	**464.34 (411.72, 516.96)**	**<.001**
**Premature death, y lost/1000 people**	**7.52 (5.99, 9.04)**	**<.001**	**16.62 (14.70, 18.54)**	**<.001**	**25.06 (22.41, 27.72)**	**<.001**
Age-adjusted mortality by cause of death /100,000 person-years						
**All cause**	**63.50 (54.43, 72.58)**	**<.001**	**118.29 (106.87, 129.71)**	**<.001**	**177.70 (161.90, 193.51)**	**<.001**
**Malignant cancers**	**11.10 (9.39, 12.81)**	**<.001**	**18.96 (16.81, 21.11)**	**<.001**	**28.71 (25.74, 31.69)**	**<.001**
**Lung and bronchus**	**3.61 (2.88, 4.34)**	**<.001**	**7.27 (6.35, 8.18)**	**<.001**	**11.33 (10.07, 12.60)**	**<.001**
**Prostate**	**1.57 (1.05, 2.08)**	**<.001**	**1.81 (1.16, 2.46)**	**<.001**	**3.37 (2.45, 4.29)**	**<.001**
**Female breast**	**1.11 (0.71, 1.51)**	**<.001**	**1.54 (1.04, 2.04)**	**<.001**	**2.63 (1.92, 3.34)**	**<.001**
**Colon and rectum**	**0.87 (0.59, 1.15)**	**<.001**	**1.46 (1.11, 1.81)**	**<.001**	**2.62 (2.13, 3.11)**	**<.001**
**Pancreas**	**0.46 (0.26, 0.66)**	**<.001**	**0.74 (0.48, 0.99)**	**<.001**	**1.34 (0.99, 1.70)**	**<.001**
**Heart disease**	**8.32 (5.28, 11.35)**	**<.001**	**19.53 (15.71, 23.35)**	**<.001**	**35.45 (30.17, 40.73)**	**<.001**
**Accidents and adverse effects**	**2.55 (1.18, 3.93)**	**<.001**	**7.03 (5.30, 8.76)**	**<.001**	**7.69 (5.30, 10.08)**	**<.001**
**Suicide and self-inflicted injury**	**0.58 (0.18, 0.99)**	**0.005**	**1.33 (0.83, 1.84)**	**<.001**	**1.69 (0.98, 2.40)**	**<.001**
**Chronic liver disease and cirrhosis**	**0.89 (0.41, 1.37)**	**<.001**	**2.24 (1.64, 2.84)**	**<.001**	**2.84 (2.01, 3.67)**	**<.001**
**Alzheimer disease**	**3.31 (2.19, 4.43)**	**<.001**	**4.84 (3.43, 6.25)**	**<.001**	**7.67 (5.71, 9.62)**	**<.001**
**Diabetes**	**2.73 (1.88, 3.58)**	**<.001**	**4.85 (3.79, 5.91)**	**<.001**	**8.74 (7.26, 10.21)**	**<.001**
**Chronic obstructive pulmonary disease and allied conditions**	**5.11 (4.03, 6.19)**	**<.001**	**10.58 (9.21, 11.94)**	**<.001**	**17.39 (15.50, 19.27)**	**<.001**
**Cerebrovascular diseases**	**4.28 (3.43, 5.13)**	**<.001**	**6.22 (5.15, 7.29)**	**<.001**	**10.47 (8.99, 11.95)**	**<.001**
**Homicide and legal intervention**	**1.92 (1.20, 2.65)**	**<.001**	**4.01 (3.09, 4.92)**	**<.001**	**4.71 (3.43, 6.00)**	**<.001**
**Septicemia**	**0.85 (0.36, 1.34)**	**<.001**	**1.68 (1.06, 2.30)**	**<.001**	**2.58 (1.72, 3.43)**	**<.001**
**Nephritis, nephrotic syndrome, and nephrosis**	**2.35 (1.84, 2.86)**	**<.001**	**3.63 (2.99, 4.27)**	**<.001**	**4.39 (3.50, 5.27)**	**<.001**
**Hypertension without heart disease**	0.26 (-0.27, 0.79)	0.34	**0.89 (0.22, 1.56)**	**0.009**	**2.57 (1.63, 3.51)**	**<.001**

Data sources include Debt in America Project of the Urban Institute, American Community Survey, Behavioral Risk Factor Surveillance System, and National Center for Health Statistics (Appendix Table 1).

Models controlled for county-level % of non-Hispanic White, % less than high school graduate, % uninsured, % unemployed, and metropolitan statistical area status, and were weighted by population size, with standard errors clustered at the state level.
[1] P-values are from T-tests.

© 2024 Han X et al. *JAMA Network Open.*

eTable 5. Association of Quartiles of County-Level Median Medical Debt With Health Status, Premature Death, and Age-Adjusted Mortality in the US

Health outcome	Q2 (vs. Q1)		Q3 (vs. Q1)		Q4 (vs. Q1)	
	Coefficient	P-value[3]	Coefficient	P-value[3]	Coefficient	P-value[3]
**Poor physical health during past 30 d, d/1000 people**	**42.13 (8.07, 76.20)**	**0.02**	**77.32 (35.10, 119.54)**	**<.001**	**71.82 (17.86, 125.78)**	**0.009**
**Poor mental health during past 30 d, d/1000 people**	28.85 (-3.74, 61.44)	0.08	36.75 (-3.66, 77.16)	0.07	**57.43 (5.79, 109.07)**	**0.029**
**Premature death, y lost/1000 people**	**2.82 (1.26, 4.39)**	**<.001**	**3.14 (1.20, 5.09)**	**0.002**	**6.50 (4.02, 8.98)**	**<.001**
Age-adjusted mortality by cause of death /100,000 person-years						
**All cause**	**22.95 (13.27, 32.63)**	**<.001**	**36.16 (24.17, 48.16)**	**<.001**	**37.58 (22.25, 52.91)**	**<.001**
**Malignant cancers**	**3.65 (1.92, 5.38)**	**<.001**	**7.49 (5.34, 9.63)**	**<.001**	**6.27 (3.53, 9.01)**	**<.001**
**Lung and bronchus**	**1.96 (1.23, 2.69)**	**<.001**	**3.08 (2.17, 3.98)**	**<.001**	**2.71 (1.55, 3.87)**	**<.001**
**Prostate**	0.09 (-0.36, 0.53)	0.70	**0.82 (0.27, 1.37)**	**0.004**	0.52 (-0.19, 1.23)	0.15
**Female breast**	0.12 (-0.22, 0.47)	0.48	**0.72 (0.29, 1.15)**	**0.001**	0.40 (-0.15, 0.95)	0.16
**Colon and rectum**	0.06 (-0.18, 0.31)	0.62	**0.49 (0.19, 0.80)**	**0.002**	0.34 (-0.05, 0.73)	0.09
**Pancreas**	0.08 (-0.10, 0.26)	0.38	**0.30 (0.08, 0.52)**	**0.008**	0.24 (-0.04, 0.52)	0.09
**Heart disease**	1.45 (-1.63, 4.53)	0.36	**6.13 (2.31, 9.94)**	**0.002**	**6.14 (1.26, 11.01)**	**0.01**
**Accidents and adverse effects**	**1.65 (0.29, 3.01)**	**0.017**	-0.16 (-1.84, 1.52)	0.85	1.80 (-0.35, 3.95)	0.10
**Suicide and self-inflicted injury**	0.01 (-0.33, 0.34)	0.97	0.21 (-0.21, 0.63)	0.33	**1.06 (0.52, 1.59)**	**<.001**
**Chronic liver disease and cirrhosis**	0.29 (-0.12, 0.71)	0.17	-0.47 (-0.98, 0.04)	0.07	**0.76 (0.10, 1.42)**	**0.023**
**Alzheimer disease**	0.38 (-0.69, 1.45)	0.49	**2.46 (1.13, 3.79)**	**<.001**	0.85 (-0.85, 2.55)	0.33
**Diabetes**	**1.08 (0.26, 1.90)**	**0.010**	**1.99 (0.97, 3.00)**	**<.001**	**2.29 (1.00, 3.59)**	**<.001**
**Chronic obstructive pulmonary disease and allied conditions**	**1.96 (0.84, 3.07)**	**<.001**	**4.10 (2.72, 5.48)**	**<.001**	**3.36 (1.59, 5.13)**	**<.001**
**Cerebrovascular diseases**	**1.64 (0.78, 2.50)**	**<.001**	**2.79 (1.72, 3.85)**	**<.001**	**1.39 (0.03, 2.75)**	**0.045**
**Homicide and legal intervention**	0.63 (-0.003, 1.27)	0.05	**0.79 (0.002, 1.58)**	**0.050**	**1.21 (0.17, 2.25)**	**0.023**
**Septicemia**	**1.01 (0.58, 1.45)**	**<.001**	**0.68 (0.13, 1.22)**	**0.014**	**0.95 (0.25, 1.64)**	**0.008**
**Nephritis, nephrotic syndrome, and nephrosis**	**1.29 (0.82, 1.76)**	**<.001**	**1.67 (1.09, 2.25)**	**<.001**	**1.22 (0.48, 1.97)**	**0.001**
**Hypertension without heart disease**	0.10 (-0.35, 0.55)	0.69	0.34 (-0.22, 0.90)	0.24	0.44 (-0.29, 1.16)	0.24

Data sources include Debt in America Project of the Urban Institute, American Community Survey, Behavioral Risk Factor Surveillance System, and National Center for Health Statistics (Appendix Table 1).

Models controlled for county-level % of non-Hispanic White, % less than high school graduate, % uninsured, % unemployed, and metropolitan statistical area status, and were weighted by population size, with standard errors clustered at the state level.
[1] P-values are from T-tests.

© 2024 Han X et al. *JAMA Network Open.*

eTable 6. County-Level Debt by Sociodemographic Characteristics in the US, 2018

	Share of people with any debt (N=2943)			Median debt among people with debt ($) (N=2365)		
	Mean	SD	P-value[1]	Mean	SD	P-value[1]
**Overall**	32.7%	11.5%		1674.5	448.3	
**MSA status**			0.002			0.46
**Metropolitan**	31.9%	10.3%		1681.8	389.4	
**Non-metropolitan**	33.3%	12.2%		1668.2	493.1	
**% NH White**			<.001			<.001
**Q1: 2.822-65.609**	42.1%	10.6%		1723.7	437.0	
**Q2: 65.610-84.891**	33.2%	9.9%		1693.1	417.7	
**Q3: 84.897-93.437**	27.3%	8.9%		1670.6	468.5	
**Q4: 93.439-98.390**	28.3%	10.2%		1585.5	468.2	
**% NH Black**			<.001			0.70
**Q1: 0.129-1.091**	26.4%	10.8%		1689.2	558.4	
**Q2: 1.092-2.709**	28.9%	9.9%		1671.0	489.0	
**Q3: 2.710-10.775**	33.1%	9.8%		1660.1	403.8	
**Q4: 10.776-85.962**	41.5%	9.3%		1683.0	387.3	
**% Hispanic**			<.001			<.001
**Q1: 0.577-2.308**	32.9%	11.3%		1601.4	479.3	
**Q2: 2.309-4.286**	31.3%	11.8%		1662.6	429.2	
**Q3: 4.287-9.712**	32.0%	11.2%		1694.4	410.7	
**Q4: 9.713-96.262**	34.8%	11.4%		1730.8	463.4	
**% API**			<.001			0.007
**Q1: 0.067-0.486**	34.7%	12.1%		1631.3	517.7	
**Q2: 0.486-0.757**	34.0%	12.3%		1657.5	442.7	
**Q3: 0.758-1.512**	33.3%	11.0%		1722.6	469.2	
**Q4: 1.513-69.786**	29.4%	9.8%		1670.5	381.7	
**% AIAN**			<.001			<.001
**Q1: 0.025-0.258**	33.1%	11.3%		1573.0	403.6	
**Q2: 0.258-0.403**	34.4%	11.7%		1652.5	415.0	
**Q3: 0.403-0.847**	33.3%	11.7%		1728.2	440.9	
**Q4: 0.848-84.855**	29.9%	10.7%		1780.4	523.0	
**% Foreign born**			0.09			<.001
**Q1: 0.000-1.380**	33.7%	11.7%		1603.9	484.8	
**Q2: 1.381-2.730**	32.4%	11.6%		1687.6	456.3	
**Q3: 2.731-5.670**	32.7%	11.2%		1721.3	447.6	
**Q4: 5.671-53.720**	32.2%	11.5%		1668.0	407.1	
**% 65 years or older**			<.001			0.31
**Q1: 3.200-15.800**	34.0%	12.0%		1700.7	447.5	
**Q2: 15.801-18.440**	34.9%	11.4%		1657.0	433.6	
**Q3: 18.441-21.220**	32.8%	11.0%		1667.1	437.2	
**Q4: 21.221-56.710**	28.7%	10.5%		1670.9	490.1	
**% Less than high school graduate**			<.001			0.11

© 2024 Han X et al. *JAMA Network Open.*

**Q1: 1.120-8.450**	21.7%	6.5%		1691.6	481.8	
**Q2: 8.451-11.720**	28.8%	8.1%		1654.1	403.6	
**Q3: 11.721-16.650**	36.5%	8.5%		1702.4	438.5	
**Q4: 16.651-46.690**	43.4%	9.3%		1651.5	469.3	
**% Below poverty**			<.001			0.14
**Q1: 2.770-10.610**	22.9%	7.6%		1657.5	461.5	
**Q2: 10.611-14.180**	28.6%	8.8%		1707.5	457.8	
**Q3: 14.181-18.460**	35.5%	9.1%		1680.2	419.2	
**Q4: 18.461-55.450**	43.0%	9.2%		1653.0	456.3	
**% Uninsured**			<.001			<.001
**Q1: 2.105-7.311**	23.6%	8.3%		1603.4	412.5	
**Q2: 7.312-10.315**	30.7%	9.4%		1613.5	431.6	
**Q3: 10.316-13.730**	35.7%	9.8%		1705.8	441.8	
**Q4: 13.731-43.798**	41.3%	10.3%		1772.6	483.2	
**% Unemployed**			<.001			0.05
**Q1: 0.000-3.620**	23.6%	10.0%		1714.9	543.3	
**Q2: 3.621-4.930**	30.3%	9.3%		1640.2	393.3	
**Q3: 4.931-6.480**	34.9%	9.3%		1671.0	425.9	
**Q4: 6.481-23.370**	41.2%	10.0%		1690.8	463.6	

Data sources include Debt in America Project of the Urban Institute and American Community Survey (Appendix Table 1). The measure of debt in this Table includes student loan debt in default, auto/retail loan delinquency, credit card debt delinquency, and medical debt in collections.

Abbreviations: MSA: metropolitan statistical area; NH: non-Hispanic; API= Asian pacific islander; AIAN=American Indian and Alaska Native; Q: quartile; SD: standard deviation.

The range of percentages in each quarter of the counties are presented in 1st column.

[1] P-values are from T-tests.

© 2024 Han X et al. *JAMA Network Open.*

eTable 7. Association of County-Level Share of Population With Any Debt With Health Status, Premature Death, and Age-Adjusted Mortality in the US

Health outcome	N	Mean	SD	Crude model[1]		Adjusted model [2]	
				Coefficient	P-value[3]	Coefficient	P-value[3]
**Poor physical health during past 30 d, d/1000 people**	2943	4413.2	766.4	**55.0 (53.2, 56.7)**	**<.001**	**31.7 (30.0, 33.4)**	**<.001**
**Poor mental health during past 30 d, d/1000 people**	2943	4702.2	658.8	**38.2 (36.7, 39.8)**	**<.001**	**30.3 (28.8, 31.9)**	**<.001**
**Premature death, y lost/1000 people**	2837	85.2	25.3	**1.84 (1.78, 1.91)**	**<.001**	**1.72 (1.64, 1.79)**	**<.001**
Age-adjusted mortality by cause of death /100,000 person-years							
**All cause**	2943	824.6	146.5	**10.19 (9.81, 10.57)**	**<.001**	**11.24 (10.82, 11.67)**	**<.001**
**Malignant cancers**	2943	166.8	27.4	**1.49 (1.41, 1.56)**	**<.001**	**1.75 (1.67, 1.84)**	**<.001**
**Lung and bronchus**	2888	44.3	12.9	**0.53 (0.49, 0.57)**	**<.001**	**0.71 (0.67, 0.75)**	**<.001**
**Prostate**	2020	20.7	6.5	**0.23 (0.21, 0.25)**	**<.001**	**0.21 (0.18, 0.24)**	**<.001**
**Female breast**	2201	21.2	5.3	**0.16 (0.15, 0.18)**	**<.001**	**0.16 (0.14, 0.19)**	**<.001**
**Colon and rectum**	2538	15.8	4.5	**0.17 (0.16, 0.19)**	**<.001**	**0.14 (0.13, 0.16)**	**<.001**
**Pancreas**	2312	11.8	2.8	**0.05 (0.04, 0.06)**	**<.001**	**0.07 (0.06, 0.09)**	**<.001**
**Heart disease**	2943	187.1	48.2	**2.64 (2.52, 2.77)**	**<.001**	**2.20 (2.03, 2.37)**	**<.001**
**Accidents and adverse effects**	2917	58.4	18.4	**0.79 (0.73, 0.85)**	**<.001**	**0.83 (0.75, 0.90)**	**<.001**
**Suicide and self-inflicted injury**	2388	18.7	7.1	**0.07 (0.04, 0.09)**	**<.001**	**0.13 (0.10, 0.15)**	**<.001**
**Chronic liver disease and cirrhosis**	2227	13.6	7.1	**0.26 (0.24, 0.28)**	**<.001**	**0.18 (0.16, 0.21)**	**<.001**
**Alzheimer disease**	2741	34.1	14.2	**0.14 (0.09, 0.19)**	**<.001**	**0.36 (0.30, 0.43)**	**<.001**
**Diabetes**	2744	26.6	12.1	**0.60 (0.56, 0.63)**	**<.001**	**0.44 (0.39, 0.49)**	**<.001**
**Chronic obstructive pulmonary disease and allied conditions**	2921	52.1	17.7	**0.75 (0.70, 0.81)**	**<.001**	**0.98 (0.92, 1.04)**	**<.001**
**Cerebrovascular diseases**	2884	40.4	10.6	**0.42 (0.39, 0.46)**	**<.001**	**0.56 (0.51, 0.61)**	**<.001**
**Homicide and legal intervention**	1123	8.1	6.6	**0.43 (0.40, 0.46)**	**<.001**	**0.38 (0.34, 0.43)**	**<.001**
**Septicemia**	2244	13.5	6.1	**0.29 (0.27, 0.31)**	**<.001**	**0.25 (0.22, 0.28)**	**<.001**
**Nephritis, nephrotic syndrome, and nephrosis**	2436	16.6	7.2	**0.31 (0.29, 0.33)**	**<.001**	**0.30 (0.27, 0.33)**	**<.001**
**Hypertension without heart disease**	1973	10.6	6.8	**0.19 (0.17, 0.22)**	**<.001**	**0.14 (0.11, 0.17)**	**<.001**

Data sources include Debt in America Project of the Urban Institute, American Community Survey, Behavioral Risk Factor Surveillance System, and National Center for Health Statistics (Appendix Table 1). The measure of debt in this Table includes student loan debt in default, auto/retail loan delinquency, credit card debt delinquency, and medical debt in collections. Number of counties in analysis (N) varies by health outcomes because county level health statistics are not provided if fewer than 10 cases.

[1] Crude models were weighted by population size, with standard errors clustered at the state level.
[2] Adjusted models controlled for county-level % of non-Hispanic White, % less than high school graduate, % uninsured, % unemployed, and metropolitan statistical area status. All models were weighted by population size, with standard errors clustered at the state level.
[3] P-values are from T-tests.

© 2024 Han X et al. *JAMA Network Open.*

eTable 8. Association of County-Level Median Debt (in 2018 Hundred Dollars) With Health Status, Premature Death, and Age-Adjusted Mortality in the US

Health outcome	N	Mean	SD	Crude model[1]		Adjusted model [2]	
				Coefficient	P-value[3]	Coefficient	P-value[3]
**Poor physical health during past 30 d, d/1000 people**	2365	4481.0	736.1	**27.9 (20.7, 35.1)**	**<.001**	**16.4 (12.6, 20.2)**	**<.001**
**Poor mental health during past 30 d, d/1000 people**	2365	4781.4	621.9	**23.8 (18.3, 29.4)**	**<.001**	**10.2 (6.5, 13.9)**	**<.001**
**Premature death, y lost/1000 people**	2364	85.6	24.6	**1.45 (1.22, 1.69)**	**<.001**	**0.84 (0.66, 1.02)**	**<.001**
Age-adjusted mortality by cause of death /100,000 person-years							
**All cause**	2365	834.9	145.2	**9.98 (8.57, 11.40)**	**<.001**	**6.45 (5.35, 7.54)**	**<.001**
**Malignant cancers**	2365	168.5	26.2	**1.74 (1.50, 1.98)**	**<.001**	**1.17 (0.97, 1.37)**	**<.001**
**Lung and bronchus**	2358	44.9	12.7	**0.67 (0.56, 0.78)**	**<.001**	**0.39 (0.30, 0.47)**	**<.001**
**Prostate**	1945	20.5	6.1	**0.14 (0.08, 0.20)**	**<.001**	**0.12 (0.06, 0.17)**	**<.001**
**Female breast**	2122	21.0	5.0	**0.13 (0.08, 0.17)**	**<.001**	**0.09 (0.04, 0.13)**	**<.001**
**Colon and rectum**	2289	15.5	4.1	**0.15 (0.11, 0.18)**	**<.001**	**0.11 (0.08, 0.14)**	**<.001**
**Pancreas**	2173	11.6	2.6	**0.07 (0.05, 0.09)**	**<.001**	**0.05 (0.03, 0.07)**	**<.001**
**Heart disease**	2365	190.3	48.5	**2.17 (1.74, 2.60)**	**<.001**	**1.47 (1.11, 1.82)**	**<.001**
**Accidents and adverse effects**	2364	58.1	17.7	**0.77 (0.59, 0.95)**	**<.001**	**0.30 (0.14, 0.45)**	**<.001**
**Suicide and self-inflicted injury**	2205	17.9	5.9	**0.32 (0.26, 0.37)**	**<.001**	**0.15 (0.11, 0.19)**	**<.001**
**Chronic liver disease and cirrhosis**	2137	13.2	6.3	**0.25 (0.19, 0.31)**	**<.001**	**0.15 (0.10, 0.20)**	**<.001**
**Alzheimer disease**	2331	34.4	13.8	**0.28 (0.15, 0.40)**	**<.001**	**0.23 (0.11, 0.36)**	**<.001**
**Diabetes**	2340	26.0	12.0	**0.42 (0.31, 0.53)**	**<.001**	**0.36 (0.26, 0.45)**	**<.001**
**Chronic obstructive pulmonary disease and allied conditions**	2362	52.4	17.6	**1.03 (0.86, 1.20)**	**<.001**	**0.62 (0.49, 0.75)**	**<.0001**
**Cerebrovascular diseases**	2362	40.8	10.3	**0.35 (0.25, 0.45)**	**<.001**	**0.26 (0.16, 0.36)**	**<.001**
**Homicide and legal intervention**	1122	8.1	6.6	**0.14 (0.03, 0.25)**	**0.016**	0.06 (-0.02, 0.15)	0.16
**Septicemia**	2158	13.3	6.0	**0.21 (0.15, 0.27)**	**<.001**	**0.13 (0.08, 0.18)**	**<.001**
**Nephritis, nephrotic syndrome, and nephrosis**	2257	16.3	6.8	**0.25 (0.19, 0.31)**	**<.001**	**0.19 (0.13, 0.24)**	**<.001**
**Hypertension without heart disease**	1895	10.3	6.6	0.06 (-0.0002, 0.12)	0.05	0.05 (-0.01, 0.11)	0.10

Data sources include Debt in America Project of the Urban Institute, American Community Survey, Behavioral Risk Factor Surveillance System, and National Center for Health Statistics (Appendix Table 1). The measure of debt in this Table includes student loan debt in default, auto/retail loan delinquency, credit card debt delinquency, and medical debt in collections. Number of counties in analysis (N) varies by health outcomes because county level health statistics are not provided if fewer than 10 cases.

[1] Crude models were weighted by population size, with standard errors clustered at the state level.

[2] Adjusted models controlled for county-level % of non-Hispanic White, % less than high school graduate, % uninsured, % unemployed, and metropolitan statistical area status. All models were weighted by population size, with standard errors clustered at the state level.

[3] P-values are from T-tests.

© 2024 Han X et al. *JAMA Network Open.*

eTable 9. Association of County-Level Debt and Age-Adjusted Mortality Rate (per 100 000 Person-Years) by Sex in the US

	Any debt				Median debt (2018 USD $100)			
	Men		Women		Men		Women	
Cause of mortality	Coefficient	P-value[1]	Coefficient	P-value[1]	Coefficient	P-value[1]	Coefficient	P-value[1]
All cause	14.25 (13.69, 14.80)	<.001	9.40 (9.03, 9.77)	<.001	7.92 (6.52, 9.32)	<.001	5.32 (4.38, 6.25)	<.001
Malignant cancers	2.40 (2.27, 2.52)	<.001	1.39 (1.31, 1.47)	<.001	1.49 (1.21, 1.77)	<.001	0.93 (0.75, 1.10)	<.001
Lung and bronchus	0.94 (0.89, 1.00)	<.001	0.57 (0.53, 0.61)	<.001	0.48 (0.36, 0.60)	<.001	0.32 (0.24, 0.40)	<.001
Prostate	0.22 (0.19, 0.25)	<.001	-	-	0.12 (0.06, 0.17)	<.001	-	-
Female breast	-	-	0.16 (0.13, 0.18)	<.001	-	-	0.09 (0.05, 0.13)	<.001
Colon and rectum	0.19 (0.16, 0.21)	<.001	0.12 (0.10, 0.14)	<.001	0.12 (0.08, 0.17)	<.001	0.08 (0.05, 0.11)	<.001
Pancreas	0.08 (0.06, 0.10)	<.001	0.07 (0.05, 0.08)	<.001	0.07 (0.04, 0.11)	<.001	0.03 (0.00, 0.06)	0.022
Heart disease	3.00 (2.79, 3.22)	<.001	1.75 (1.60, 1.90)	<.001	1.92 (1.48, 2.37)	<.001	1.12 (0.82, 1.43)	<.001
Accidents and adverse effects	1.10 (0.99, 1.21)	<.001	0.56 (0.50, 0.62)	<.001	0.43 (0.21, 0.65)	<.001	0.24 (0.12, 0.35)	<.001
Suicide and self-inflicted injury	0.24 (0.20, 0.28)	<.001	0.03 (0.01, 0.05)	0.009	0.25 (0.18, 0.32)	<.001	0.05 (0.01, 0.10)	0.01
Chronic liver disease and cirrhosis	0.22 (0.18, 0.27)	<.001	0.12 (0.09, 0.15)	<.001	0.21 (0.13, 0.28)	<.001	0.14 (0.09, 0.20)	<.001
Alzheimer disease	0.36 (0.30, 0.43)	<.001	0.39 (0.31, 0.47)	<.00	0.19 (0.07, 0.31)	0.002	0.22 (0.07, 0.36)	0.003
Diabetes	0.53 (0.47, 0.60)	<.001	0.38 (0.34, 0.43)	<.0001	0.45 (0.32, 0.57)	<.001	0.27 (0.18, 0.36)	<.001
Chronic obstructive pulmonary disease and allied conditions	1.13 (1.06, 1.20)	<.001	0.95 (0.89, 1.01)	<.001	0.70 (0.55, 0.85)	<.001	0.52 (0.39, 0.65)	<.001
Cerebrovascular diseases	0.64 (0.59, 0.70)	<.001	0.53 (0.48, 0.58)	<.001	0.30 (0.19, 0.41)	<.00	0.21 (0.11, 0.32)	<.001
Homicide and legal intervention	0.67 (0.59, 0.76)	<.001	0.12 (0.10, 0.14)	<.001	0.08 (-0.11, 0.26)	0.41	0.05 (-0.001, 0.09)	0.06
Septicemia	0.29 (0.25, 0.33)	<.001	0.23 (0.20, 0.26)	<.001	0.16 (0.08, 0.23)	<.0001	0.12 (0.06, 0.18)	<.001
Nephritis, nephrotic syndrome, and nephrosis	0.34 (0.30, 0.38)	<.001	0.28 (0.25, 0.31)	<.001	0.21 (0.13, 0.29)	<.001	0.19 (0.13, 0.25)	<.001
Hypertension without heart disease	0.14 (0.09, 0.19)	<.001	0.13 (0.09, 0.17)	<.001	0.04 (-0.04, 0.13)	0.33	0.04 (-0.03, 0.11)	0.22

Data sources include Debt in America Project of the Urban Institute, American Community Survey, Behavioral Risk Factor Surveillance System, and National Center for Health Statistics (Appendix Table 1).

Models controlled for county-level % of non-Hispanic White, % less than high school graduate, % uninsured, % unemployed, and metropolitan statistical area status. All models were weighted by population size, with standard errors clustered at the state level.

[1] P-values are from T-tests.

© 2024 Han X et al. *JAMA Network Open.*

eTable 10. Association of Quartiles of County-Level Share of Population With Any Debt With Health Status, Premature Death, and Age-Adjusted Mortality in the US

Health outcome	Q2 (vs. Q1)		Q3 (vs. Q1)		Q4 (vs. Q1)	
	Coefficient	P-value[1]	Coefficient	P-value[1]	Coefficient	P-value[1]
**Poor physical health during past 30 d, d/1000 people**	**195.28 (14.51, 24.54)**	**<.001**	**463.10 (405.33, 520.87)**	**<.001**	**698.69 (626.69, 770.69)**	**<.001**
**Poor mental health during past 30 d, d/1000 people**	**287.43 (24.06, 33.43)**	**<.001**	**534.72 (480.77, 588.67)**	**<.001**	**781.42 (714.18, 848.67)**	**<.001**
**Premature death, y lost/1000 people**	**9.14 (6.78, 11.50)**	**<.001**	**22.64 (19.93, 25.34)**	**<.001**	**38.63 (35.26, 42.00)**	**<.001**
Age-adjusted mortality by cause of death /100,000 person-years						
**All cause**	**72.60 (58.67, 86.53)**	**<.001**	**165.94 (149.93, 181.94)**	**<.001**	**259.31 (239.37, 279.26)**	**<.001**
**Malignant cancers**	**11.17 (8.48, 13.85)**	**<.001**	**25.53 (22.45, 28.60)**	**<.001**	**39.29 (35.46, 43.13)**	**<.001**
**Lung and bronchus**	**3.02 (1.88, 4.16)**	**<.001**	**9.14 (7.83, 10.45)**	**<.001**	**14.68 (13.05, 16.32)**	**<.001**
**Prostate**	**2.42 (1.58, 3.25)**	**<.001**	**3.74 (2.78, 4.70)**	**<.001**	**6.13 (4.93, 7.33)**	**<.001**
**Female breast**	**1.62 (0.95, 2.28)**	**<.001**	**2.98 (2.22, 3.75)**	**<.001**	**4.34 (3.39, 5.29)**	**<.001**
**Colon and rectum**	**0.61 (0.15, 1.06)**	**0.009**	**1.74 (1.22, 2.26)**	**<.001**	**3.12 (2.47, 3.77)**	**<.001**
**Pancreas**	**0.57 (0.23, 0.91)**	**<.001**	**1.03 (0.64, 1.41)**	**<.001**	**1.88 (1.40, 2.36)**	**<.001**
**Heart disease**	**13.41 (8.62, 18.19)**	**<.001**	**31.66 (26.15, 37.17)**	**<.001**	**49.83 (42.96, 56.69)**	**<.001**
**Accidents and adverse effects**	**4.41 (2.25, 6.57)**	**<.001**	**13.10 (10.62, 15.57)**	**<.001**	**16.88 (13.79, 19.97)**	**<.001**
**Suicide and self-inflicted injury**	**1.16 (0.52, 1.81)**	**<.001**	**2.33 (1.59, 3.07)**	**<.001**	**2.49 (1.57, 3.42)**	**<.001**
**Chronic liver disease and cirrhosis**	**1.48 (0.69, 2.26)**	**<.001**	**2.46 (1.56, 3.36)**	**<.001**	**3.85 (2.73, 4.96)**	**<.001**
**Alzheimer disease**	**7.73 (5.95, 9.51)**	**<.001**	**10.24 (8.20, 12.28)**	**<.001**	**14.20 (11.65, 16.75)**	**<.001**
**Diabetes**	**1.53 (0.15, 2.91)**	**0.029**	**4.20 (2.62, 5.77)**	**<.001**	**9.93 (7.96, 11.89)**	**<.001**
**Chronic obstructive pulmonary disease and allied conditions**	**6.94 (5.23, 8.65)**	**<.0001**	**16.19 (14.23, 18.16)**	**<.001**	**22.46 (20.01, 24.91)**	**<.001**
**Cerebrovascular diseases**	**5.29 (3.94, 6.64)**	**<.001**	**9.46 (7.91, 11.00)**	**<.001**	**15.31 (13.38, 17.24)**	**<.001**
**Homicide and legal intervention**	**1.29 (0.10, 2.49)**	**0.034**	**4.18 (2.80, 5.55)**	**<.001**	**8.24 (6.55, 9.94)**	**<.001**
**Septicemia**	0.03 (-0.76, 0.82)	0.94	**1.70 (0.80, 2.61)**	**<.001**	**3.79 (2.67, 4.91)**	**<.001**
**Nephritis, nephrotic syndrome, and nephrosis**	**1.78 (0.95, 2.62)**	**<.001**	**3.78 (2.83, 4.74)**	**<.001**	**5.93 (4.75, 7.11)**	**<.001**
**Hypertension without heart disease**	-0.37 (-1.23, 0.50)	0.404	0.60 (-0.40, 1.59)	0.24	**2.75 (1.50, 3.99)**	**<.001**

Data sources include Debt in America Project of the Urban Institute, American Community Survey, Behavioral Risk Factor Surveillance System, and National Center for Health Statistics (Appendix Table 1).

Models controlled for county-level % of non-Hispanic White, % less than high school graduate, % uninsured, % unemployed, and metropolitan statistical area status, and were weighted by population size, with standard errors clustered at the state level.

[1] P-values are from T-tests.

© 2024 Han X et al. *JAMA Network Open.*

eTable 11. Association of Quartiles of County-Level Median Debt With Health Status, Premature Death, and Age-Adjusted Mortality in the US

Health outcome	Q2 (vs. Q1)		Q3 (vs. Q1)		Q4 (vs. Q1)	
	Coefficient	P-value[1]	Coefficient	P-value[1]	Coefficient	P-value[1]
**Poor physical health during past 30 d, d/1000 people**	**63.43 (21.82, 105.04)**	**0.003**	**114.52 (74.26, 154.77)**	**<.001**	**185.90 (135.88, 235.92)**	**<.001**
**Poor mental health during past 30 d, d/1000 people**	**46.68 (6.92, 86.45)**	**0.021**	**89.04 (50.55, 127.52)**	**<.001**	**116.27 (68.44, 164.11)**	**<.001**
**Premature death, y lost/1000 people**	**5.68 (3.75, 7.60)**	**<.001**	**8.57 (6.71, 10.43)**	**<.001**	**11.69 (9.38, 14.00)**	**<.001**
Age-adjusted mortality by cause of death /100,000 person-years						
**All cause**	**39.69 (27.87, 51.50)**	**<.001**	**63.52 (52.10, 74.93)**	**<.001**	**84.80 (70.63, 98.98)**	**<.001**
**Malignant cancers**	**5.69 (3.54, 7.84)**	**<.001**	**11.17 (9.09, 13.24)**	**<.001**	**15.13 (12.55, 17.71)**	**<.001**
**Lung and bronchus**	**2.90 (2.00, 3.81)**	**<.001**	**4.71 (3.84, 5.59)**	**<.001**	**6.01 (4.92, 7.10)**	**<.001**
**Prostate**	0.35 (-0.25, 0.95)	0.25	**0.81 (0.23, 1.38)**	**0.006**	**1.14 (0.42, 1.85)**	**0.002**
**Female breast**	0.46 (-0.01, 0.92)	0.05	**0.93 (0.48, 1.38)**	**<.001**	**0.97 (0.41, 1.52)**	**<.001**
**Colon and rectum**	**0.45 (0.13, 0.77)**	**0.007**	**0.89 (0.58, 1.21)**	**<.001**	**1.40 (1.01, 1.79)**	**<.001**
**Pancreas**	0.15 (-0.09, 0.38)	0.22	**0.43 (0.20, 0.65)**	**<.001**	**0.64 (0.36, 0.92)**	**<.001**
**Heart disease**	**11.48 (7.70, 15.26)**	**<.001**	**15.21 (11.56, 18.86)**	**<.001**	**21.13 (16.59, 25.66)**	**<.001**
**Accidents and adverse effects**	**4.30 (2.62, 5.99)**	**<.001**	**5.66 (4.03, 7.28)**	**<.001**	**5.02 (3.00, 7.04)**	**<.001**
**Suicide and self-inflicted injury**	0.17 (-0.28, 0.61)	0.46	0.42 (-0.01, 0.85)	0.06	**1.38 (0.85, 1.92)**	**<.001**
**Chronic liver disease and cirrhosis**	0.30 (-0.23, 0.84)	0.27	0.50 (-0.03, 1.02)	0.06	**1.36 (0.71, 2.01)**	**<.001**
**Alzheimer disease**	1.08 (-0.26, 2.42)	0.11	**2.89 (1.59, 4.19)**	**<.001**	**3.72 (2.10, 5.33)**	**<.001**
**Diabetes**	**1.46 (0.43, 2.50)**	**0.006**	**2.40 (1.40, 3.40)**	**<.01**	**4.29 (3.05, 5.53)**	**<.001**
**Chronic obstructive pulmonary disease and allied conditions**	**2.46 (1.09, 3.84)**	**<.001**	**5.24 (3.91, 6.57)**	**<.0001**	**7.79 (6.14, 9.44)**	**<.001**
**Cerebrovascular diseases**	**1.92 (0.87, 2.98)**	**<.001**	**4.12 (3.10, 5.14)**	**<.001**	**3.12 (1.85, 4.38)**	**<.001**
**Homicide and legal intervention**	0.62 (-0.28, 1.52)	0.18	**1.20 (0.33, 2.08)**	**0.007**	**1.12 (0.03, 2.20)**	**0.044**
**Septicemia**	**0.65 (0.10, 1.21)**	**0.021**	**0.77 (0.23, 1.31)**	**0.005**	**1.61 (0.94, 2.28)**	**<.001**
**Nephritis, nephrotic syndrome, and nephrosis**	**1.14 (0.56, 1.73)**	**<.001**	**2.61 (2.05, 3.18)**	**<.001**	**2.27 (1.57, 2.98)**	**<.001**
**Hypertension without heart disease**	**0.86 (0.24, 1.47)**	**0.007**	0.20 (-0.39, 0.80)	0.50	**1.34 (0.60, 2.08)**	**<.001**

Data sources include Debt in America Project of the Urban Institute, American Community Survey, Behavioral Risk Factor Surveillance System, and National Center for Health Statistics (Appendix Table 1).

Models controlled for county-level % of non-Hispanic White, % less than high school graduate, % uninsured, % unemployed, and metropolitan statistical area status, and were weighted by population size, with standard errors clustered at the state level.

[1] P-values are from T-tests.

© 2024 Han X et al. *JAMA Network Open.*

eFigure 1. Medical Debt by County in the US, 2018

(A)

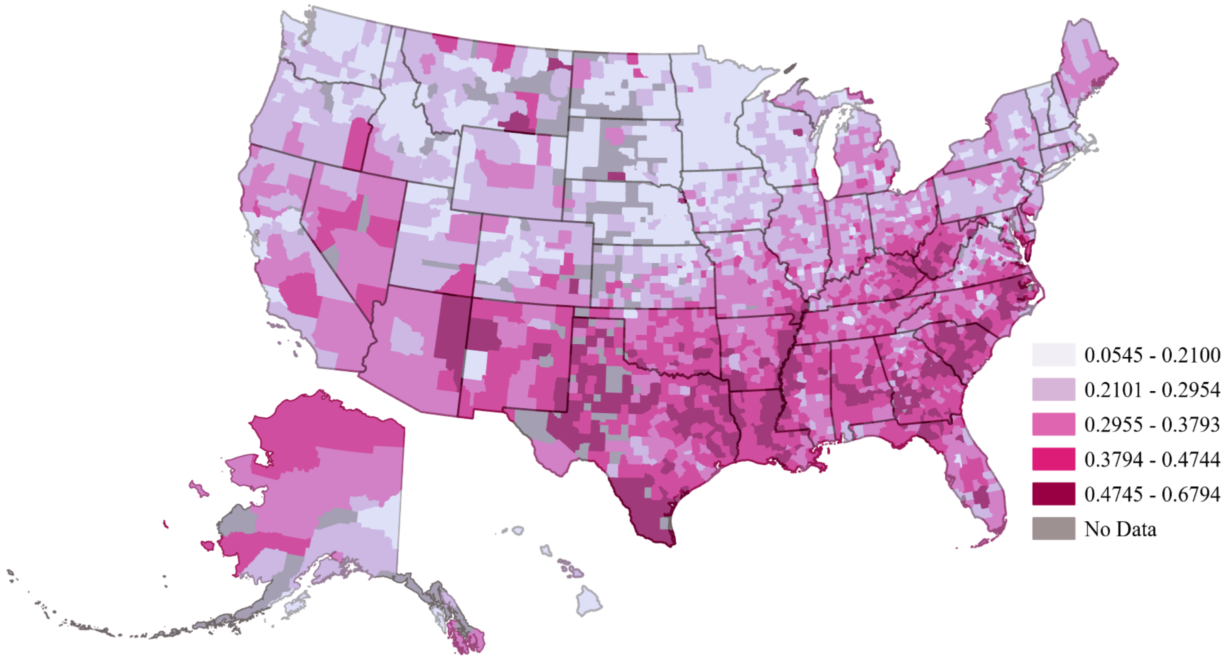

(B)

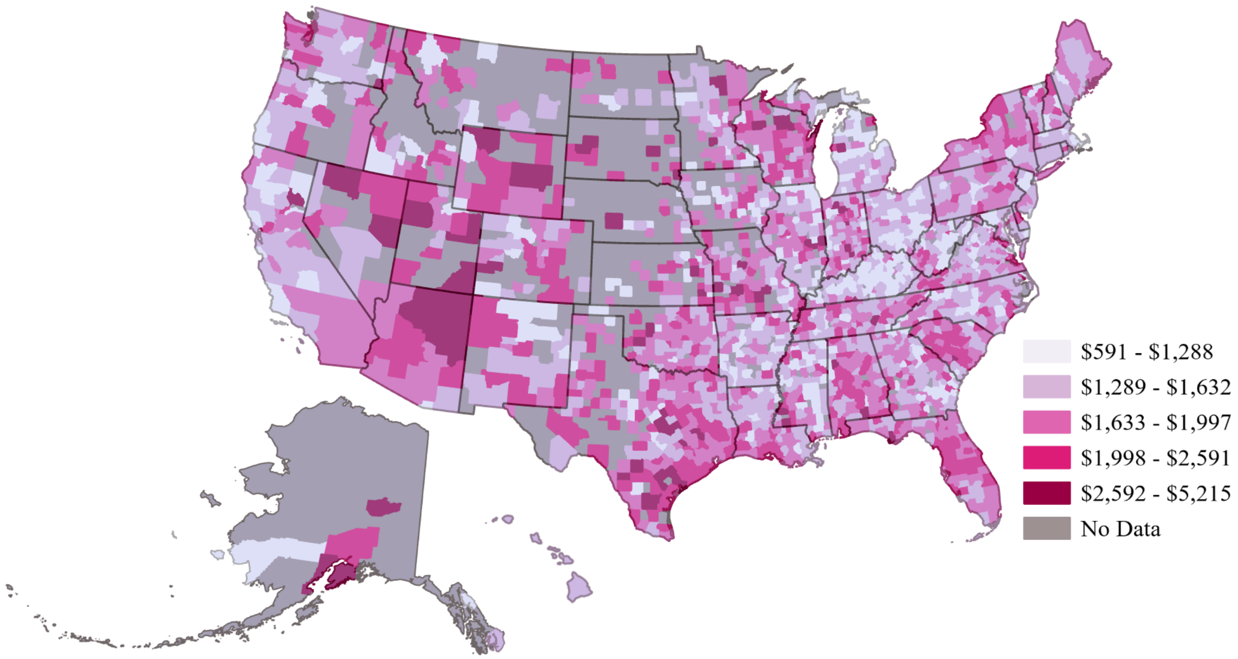

(A) Share of population with any debt in collections. (B) Median debt among people with any medical debt in collections. Data source: Debt in America Project of the Urban Institute. The measure of debt includes student loan debt in default, auto/retail loan delinquency, credit card debt delinquency, and medical debt in collections.

© 2024 Han X et al. *JAMA Network Open.*

eFigure 2. County-Level Debt and Age-Adjusted All-Cause Mortality in the US

(A)

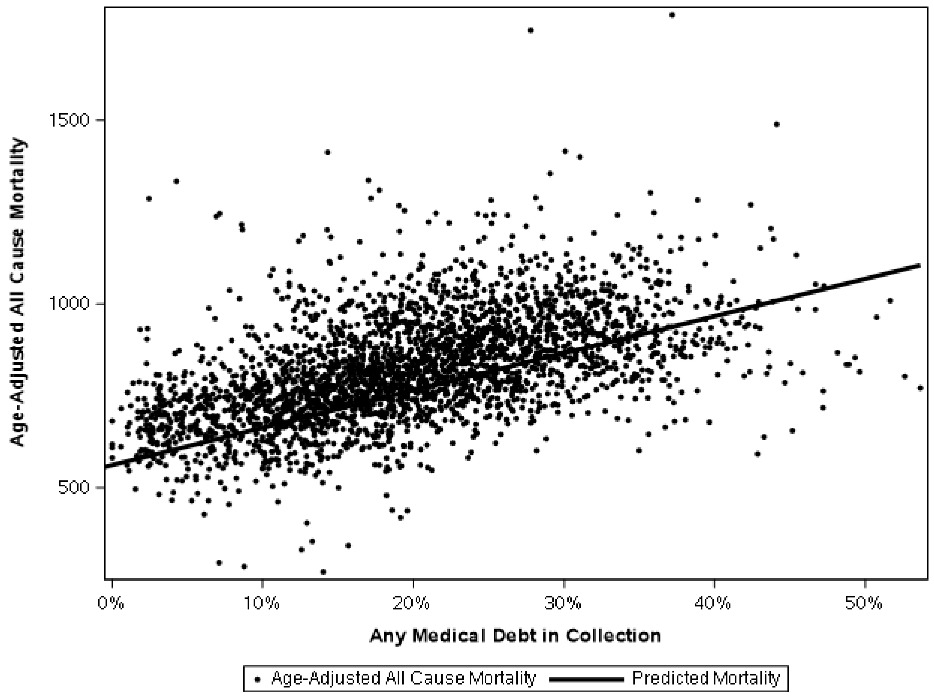

(B)

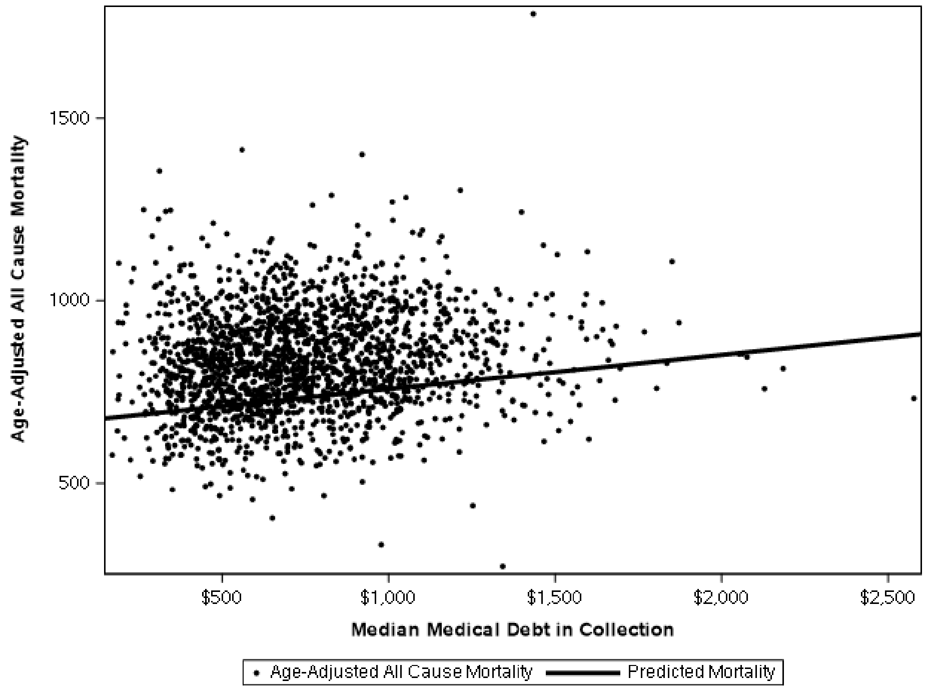

© 2024 Han X et al. *JAMA Network Open.*

(C)

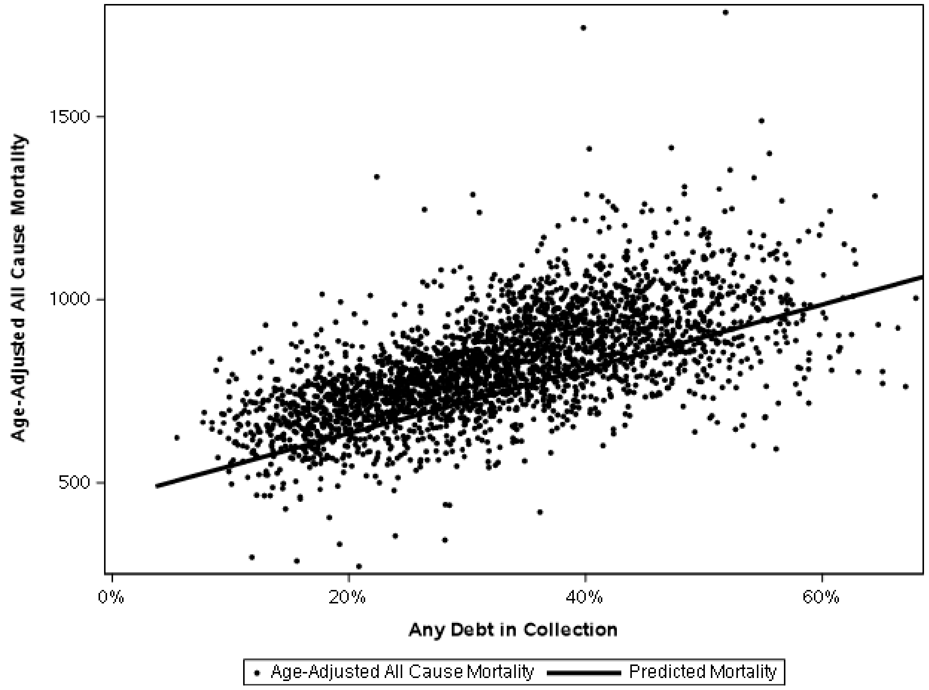

(D)

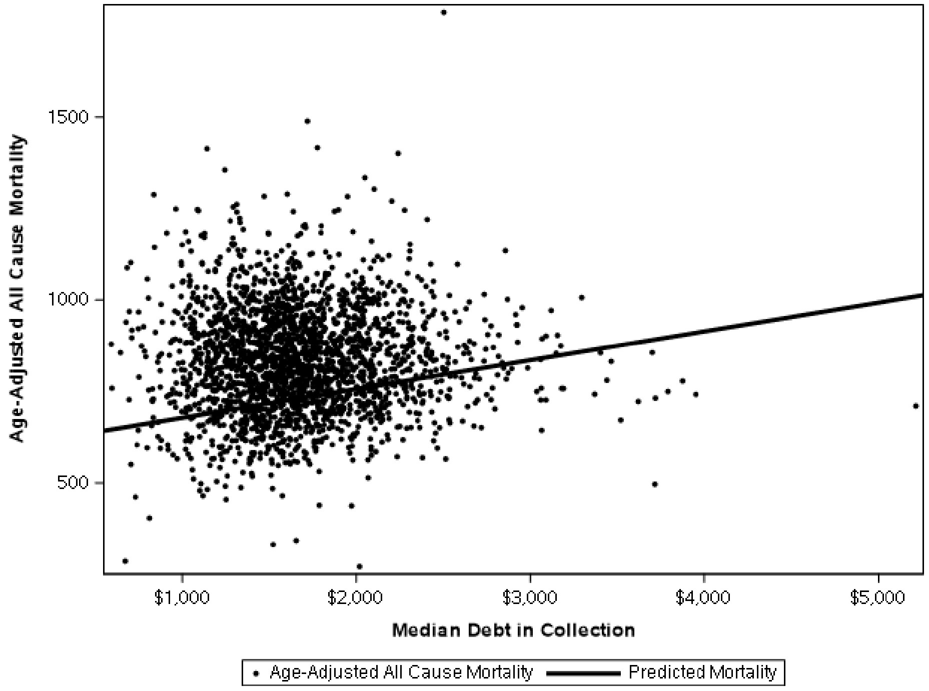

(A) Share of population with any medical debt in collections. (B) Median medical debt among people with any medical debt in collections. (C) Share of population with any debt in collections. (D) Median debt among people with any debt in collections. Data sources include Debt in America Project of the Urban Institute and National Center for Health Statistics (Appendix Table 1). The measure of debt in (C) and (D) includes student loan debt in default, auto/retail loan delinquency, credit card debt delinquency, and medical debt in collections.

© 2024 Han X et al. *JAMA Network Open.*
