# Supplementary material for: Associations of Medical Debt With Health Status, Premature Death, and Mortality in the US
Source: JAMA Netw Open. 2024 Mar 4;7(3):e2354766. doi: 10.1001/jamanetworkopen.2023.54766 (PMC10912961; doi:10.1001/jamanetworkopen.2023.54766)
Supplement: Supplement 2. — Data Sharing Statement [file jamanetwopen-e2354766-s002.pdf]

Data Sharing Statement

Han. Associations of Medical Debt With Health Status, Premature Death, and Mortality in the US. *JAMA Netw Open*. Published March 04, 2024. doi:10.1001/jamanetworkopen.2023.54766

Data
Data available: Yes
Data types: Other (please specify)
Additional Information: Data aggregated at county level
How to access data: The data underlying this article consist of publicly available data from the County Health Ranking website at https://www.countyhealthrankings.org/, and the SEER*Stat Mortality Database with a signed data use agreement through https://seer.cancer.gov/data/access.html, as well as the Urban Institute Debt in America project through a special request via externalaffairs@urban.org.
When available: With publication

Supporting Documents
Document types: None

Additional Information
Who can access the data: Anyone requesting the data
Types of analyses: For research purpose
Mechanisms of data availability: With a signed data access agreement
